# lncRNA HIF1A-AS2 acts as an oncogene to regulate malignant phenotypes in cervical cancer

**DOI:** 10.3389/fonc.2025.1530677

**Published:** 2025-02-27

**Authors:** Yang Liu, Yunyan Zhang, Cha Chen, Bhaskar Roy, Qun Li, Wei Zhang, Xuan Zhang, Jieying Pu, Yuguang Li, Yanli Liu, Huanlan Liao, Jingjing Wang, Rui Zhou, Huiyan Zhuo, Youqiang Li

**Affiliations:** ^1^ Department of Clinical Laboratory, Panyu Hexian Memorial Hospital of Guangzhou, Guangzhou, Guangdong, China; ^2^ Department of Clinical Laboratory, The Third People's Hospital of Chengdu, Chengdu, Sichuan, China; ^3^ Department of Pediatric Dentistry, Affiliated Hospital of Guangzhou Medical University, Guangzhou Key Laboratory of Basic and Applied Research of Oral Regenerative Medicine, Guangzhou, Guangdong, China; ^4^ Department of Clinical Laboratory, The Second Clinical College of Guangzhou University of Chinese Medicine, Guangzhou, Guangdong, China; ^5^ Hangzhou Institute of Medicine (HIM), Chinese Academy of Sciences, Qiantang District, Hangzhou, Zhejiang, China; ^6^ Department of Clinical Laboratory, Guangzhou Liwan District People's Hospital, Guangzhou, Guangdong, China; ^7^ College of Life Sciences, University of Chinese Academy of Sciences, Beijing, China

**Keywords:** cervical cancer, lncRNA HIF1A-AS2, MiR-34b-5p, radixin, c-Jun

## Abstract

**Background:**

Long noncoding RNAs (lncRNAs) HIF1A-AS2 is upregulated in multiple human cancers and are associated with various aspects of tumor progression. However, the molecular mechanisms of HIF1A-AS2 in cervical cancer (CC) remain largely unknown. In this study, we aim to investigate the expression pattern and signaling pathways of HIF1A-AS2 in CC.

**Methods:**

The study included a group of 20 CC patients, from whom tumor tissue specimens were collected. Additionally, three distinct CC cell lines (HeLa, SiHa, CaSki) were utilized. Quantitative real-time PCR (qRT-PCR) was used to assess the transcript levels of HIF1A-AS2 in these samples. Functional studies were performed by CCK-8, Transwell and Apoptosis assays. Databases including JASPAR, miRDB and Targetscan were used for the transcription factor or target miRNA prediction, subsequent dual luciferase activity assay, chromatin immunoprecipitation (ChIP) and Ago2 immunoprecipitation (RIP) were also adopted for validation.

**Results:**

The study demonstrated that HIF1A-AS2 expression was elevated in clinical cervical cancer specimens and cultured cell lines in comparison to normal controls. Knockdown of HIF1A-AS2 notably inhibited the proliferation and invasion of cervical cancer cells, while inducing apoptosis. In contrast, HIF1A-AS2 overexpression promoted cellular proliferation and invasion and suppressed apoptosis. It was also identified that c-Jun functions as a transcription factor, activating HIF1A-AS2 expression. Additionally, HIF1A-AS2 was found to serve as a molecular sponge for miR-34b-5p, negatively regulating its expression. Furthermore, HIF1A-AS2 controlled the expression of radixin (RDX) by sponging the miR-34b-5p pathway.

**Conclusion:**

Our findings indicate that c-Jun-activated HIF1A-AS2 acts as an oncogenic factor in CC by sponging miR-34b-5p to target radixin. These findings suggest that HIF1A-AS2 might be a viable and promising therapeutic target for cervical cancer treatment.

## Introduction

Cervical cancer (CC) is a growing global burden, particularly affecting middle-aged women in developing countries (1). In 2022, there were about 604000 new cases of CC and 342000 CC-related deaths worldwide. Compared with those in 2018, both the incidence and mortality rates were greater (2, 3). CC is the most common vaccine-preventable cancer, but the vaccine is not effective for advanced CC, and about 5% of cervical carcinomas may be unrelated to human papillomavirus (HPV) (4, 5). The traditional treatments for cervical cancer include pelvic lymphadenectomy and radiation, as well as concomitant chemotherapy, radical hysterectomy, or a combination of these treatments (6). However, for advanced cervical cancer, these traditional treatments often result in poor outcomes (7). Thus, elucidating the molecular mechanisms of CC may help in the identification of new therapeutic targets.

Long noncoding RNAs (lncRNAs) are a class of RNAs >200 nt in length that do not have the ability to encode proteins (8). As a result of technological advancements, considerable progress has been made in our understanding of lncRNAs, which were previously viewed as “junk” or “noise” RNAs (9). Emerging evidence has demonstrated that lncRNAs play important roles in regulating gene expression in various human cancers (10). The lncRNA hypoxia inducible factor 1 alpha-antisense RNA 2 (HIF1A-AS2) was first discovered in 1999 by Thrash-Bingham and Tartof, who identified a transcript that could bind complementarily with the 3’-untranslated region (UTR) of HIF1α mRNA in renal clear cell carcinoma (11). A large body of research has identified HIF1A-AS2 as a tumor-related lncRNA and acts as a tumor promoter, as demonstrated by *in vitro* and *in vivo* experiments, such as in gastric cancer (GC), breast cancer (BC), bladder cancer, osteosarcoma (OS) and renal cancer (RC) (12–16). However, to our knowledge, the dysregulated expression pattern of HIF1A-AS2 and its functions, as well as potential molecular mechanisms in CC, remain unclear.

It has been reported that lncRNA expression is regulated by transcription factors (17). The transcription factor c-Jun is a proto-oncogene that is closely related to the occurrence and development of many diseases, such as malignant tumors and cerebrovascular diseases (18). Liu Chunliang et al. reported upregulated expression of c-Jun in cervical cancer tissues (19). Our bioinformatics study revealed that the transcription factor c-Jun may be a regulator of HIF1A-AS2. Numerous previous studies have focused on the downstream molecular interactions of HIF1A-AS2, but few studies have explored how the expression of HIF1A-AS2 increases. In this study, we investigated how the transcription factor c-Jun governs the modulation of HIF1A-AS2 expression.

The critical target miRNAs of HIF1A-AS2 were further explored. In 2011, Salmena L et al. first proposed a competing endogenous RNA hypothesis (ceRNA hypothesis) (20), which explains how messenger RNAs (mRNAs) and long noncoding RNAs “talk” to each other using microRNA (miRNAs) as a means of communication. To elaborate, lncRNAs can modulate the expression of mRNAs by acting as molecular sponges for miRNAs (21). On the basis of these findings, we first predicted the target miRNAs of HIF1A-AS2 via bioinformatics. The results showed that miR-34b-5p may participate in the ceRNA process. In addition, Córdova-Rivas S et al. reported that downregulated miR-34b-5p promoted cervical cancer cell proliferation (22). According to the ceRNA hypothesis, increased HIF1A-AS2 may sponge miR-34b-5p, thereby downregulating the expression of miR-34b-5p. On the basis of the ceRNA hypothesis, we predicted the target mRNAs of miR-34b-5p. The results showed that radixin may be a target. In addition, there is evidence that radixin functions as an oncogene in diverse types of carcinogenesis (23). Ou R et al. reported that the Hpv16 E7 protein induced the upregulation of KDM2A and promoted the progression of cervical cancer by regulating the miR-132-radixin pathway (23). These findings suggest that the upregulation of radixin can promote the development of cervical cancer. In this study, we found that HIF1A-AS2 was highly expressed in CC cell lines; more importantly, increased expression of HIF1A-AS2 was also detected in CC patient cancer tissues. Moreover, we systematically studied the underlying mechanism, including the upstream and downstream regulators of HIF1A-AS2.

## Materials and methods

### Bioinformatics analysis

The PROMO database (http://alggen.lsi.upc.es/cgi-bin/promo_v3/promo/promoinit.cgi?dirDB=TF8.3) was used to predict the upstream transcription factors of HIF1A-AS2, and the promoter sequence of HIF1A-AS2 used is presented in **Supplementary Table SI**. The binding sites of the transcription factors c-Jun and HIF1A-AS2 were predicted via the JASPAR database (https://jaspar.genereg.net/analysis). miRDB (http://mirdb.org/) and TargetScan (https://www.targetscan.org/vert_71/) were used to predict the target miRNAs of HIF1A-AS2 and the target genes of miR-34b-5p and potential binding sites. All the predicted data are presented in **Supplementary Table SII**-**Supplementary Table SIV**.

### Clinical sample collection

Cervical cancer tissues and paired adjacent normal tissues (n = 20) were collected from The Affiliated Hexian Memorial Hospital of Southern Medical University. The median age was 50 years and the range was 36–66 years. The clinical characteristics of CC patients are presented in **Supplementary Table SV**. All resected tissue samples were frozen immediately in liquid nitrogen after collection. All enrolled patients provided informed consent before inclusion.

### Cell lines and cell culture

All the cell lines used were obtained from iCell Bioscience Inc. (Shanghai, China), including one human immortalized epidermal cell line (HaCat), one human embryonic kidney cell line (293T), and three cervical cancer cell lines (HeLa, SiHa, CaSki). All the cell lines passed the STR authentication report. HeLa, SiHa, HaCat and 293T cells were cultured in Dulbecco’s modified Eagle’s medium (DMEM) (Gibco, USA) supplemented with 10% fetal bovine serum (Gibco, USA). CaSki cells were cultured in RPMI 1640 with 10% fetal bovine serum. All the cells were cultured at 37°C in a humidified incubator (Eppendorf, German) with 5% CO_2._ When the cell density reached 80%, the cells were harvested for subsequent experiments.

### Adenovirus, siRNA, and plasmids

Adenoviruses for the overexpression of HIF1A-AS2 (adv-HIF1A-AS2) and the control adenovirus vector (adv-NC) were constructed by Hanbio Company (Shanghai, China), and small interfering RNAs (siRNAs) for the knockdown of HIF1A-AS2 (si-HIF1A-AS2, 5’GAGTTGGAGGTGTTGAAGCAAATAT 3’), the negative control siRNA (si-NC), the miR-34b-5p mimic (ATCCGTCACAGTAATCGACTAAC), the negative control miRNA (miR-NC) and the miR-34b-5p inhibitor (UAGGCAGUGUCAUUAGCUGAUUG) were purchased from Ribo (Guangzhou, China). The sequence of si-NC and miR-NC were not provided because it involves the company’s confidential information. The pcDNA3.1 plasmid was used to overexpress the transcription factor c-Jun, and the pmirGLO, pGL3-basic, pGL3-P1, pGL3-P2-MUT and pRL-TK (internal reference) plasmids were used for the dual-luciferase reporter assay.

### Cell transfection

All the siRNAs (50 nM) and miRNA mimics (50 nM) were transfected for 6 h using 1 µL of Lipofectamine 2000 reagent (Invitrogen, USA). The recombinant adenovirus vector (adv-HIF1A-AS2, MOI = 50) and control adenovirus vector (adv-NC, MOI = 50) were used to infect cells according to the manufacturer’s protocol.

### Quantitative real-time PCR

Total RNA was extracted from tissues with an RNA isolation Kit (Omega, USA), and cell total RNA was extracted with TRIzol reagent (AG, Hunan, China). Using NanoDrop 2000 (Thermo Fisher Scientific, USA), the quality and quantity of RNA were measured. The Primer Script RT Kit (AG, Hunan, China) was applied for reverse-transcription of 1µg total RNA into cDNA, the reverse transcription condition was set to 37°C for 15min, 85°C for 5s. The expression of lncRNA and mRNA was measured using pre-mix SYBR (AG, Hunan, China) by ABI ViiA7 system (Thermo Fisher Scientific, USA). The amplification conditions: 95°C predegeneration for 30s; 95°C denature for 5s; 60°C anneal for 30s; the denature and anneal processes lasts for 40 cycles.

miRNAs were extracted from cells with a miRNA isolation kit (Omega, USA), and 10 ng of RNA was reverse-transcribed into cDNA using TaqMan™ MicroRNA Reverse Transcription Kit (Thermo Fisher Scientific, USA), Quantitative Real-time PCR was performed by Taqman™ probes (Thermo Fisher Scientific, USA). The expression level of HIF1A-AS2 was normalized to the expression levels of β-actin, and the expression of miRNA was normalized to the expression levels of U6, calculated by 2^- ∆∆CT^. The sequences for all primers in this study were listed in **Supplementary Table SVI**.

### Chromatin immunoprecipitation assays

HeLa cells were crosslinked with 1% formaldehyde for 10 min at room temperature and quenched with 0.125 M glycine for 5 min. The cells were subjected to multiple washes with PBS and subsequently lysed for 10 min in lysis buffer composed of 50 mM HEPES, 150 mM NaCl, 1 mM EDTA, 0.1% SDS, 0.1% sodium deoxycholate, and 1% Triton X-100 supplemented with a protease inhibitor cocktail. After centrifugation, the pellet was lysed in lysis buffer and subjected to sonication. The sonication conditions were as follows: power 20%, ultrasonic shock for 2 s, gap for 3 s, and ice bath cooling for 5–15 min. The chromosome fragments are in the range of about 0.1–0.5 kb. The disposed chromatin was subjected to immunoprecipitation with a normal IgG antibody (ab172730, Abcam, USA) as a negative control (NC) and an antibody (9165S, CST, USA) against c-JUN as a positive control and then incubated at 4°C overnight. The mixture was captured by protein-G magnetic beads. After immunoprecipitation, the protein–DNA crosslinks were reversed, the DNA was purified, and the precipitated DNA was subjected to qPCR. The six pairs of primers used are listed in **Supplementary Table SVI**.

### Cell counting kit-8 assay

SiHa and HeLa cells were plated in 6-well plates and then transfected with si-HIF1A-AS2 or si-NC to knockdown the expression of HIF1A-AS2, and the concentrations of si-HIF1A-AS2 and si-NC were both 50 nM. Adv-HIF1A-AS2 and adv-NC were used to overexpress HIF1A-AS2, and viruses were used at a multiplicity of infection (MOI) of 50, when the cell density reached 60%. After 48 h, the transfected cells were harvested and then seeded into 96-well plates at a concentration of 2000 cells/well. Then, cell viability was assessed at different time points (0, 24, 48, and 72 h) by adding 10 µL of CCK-8 reagent (Dojindo, Japan) to each well. After incubation at 37°C for 2 h, cell viability was determined by measuring the OD value at 450 nm via a microplate reader (Biotek Synergy H1, USA).

### Transwell invasion assays

SiHa and HeLa cells were plated in 6-well plates and transfected with si-HIF1A-AS2, si-NC, or adv-HIF1A-AS2, adv-NC. After 48 h, the transfected cells were collected and resuspended in serum-free DMEM. Then, 4 × 10^4^ cells transfected with adv-HIF1A-AS2 or adv-NC, 8 × 10^4^ cells transfected with si-HIF1A-AS2 or si-NCwere seeded into the upper chambers of a prepacked Matrigel 24-well Transwell plate (Corning, USA), and 700 µL of medium containing 10% fetal bovine serum (10% DMEM) was added to the lower chambers. After incubation for 48 h, the cells in the upper chamber were removed with a cotton swab, and the cells on the lower membrane were fixed with 4% paraformaldehyde and stained with 1% crystal violet solution (Beyotime, Beijing, China). The cells in three randomly selected fields were counted and analyzed.

### Cell apoptosis assays

After incubation for 48 h, the transfected SiHa and HeLa cells were harvested. Then, the cells were washed twice with precooled PBS. The cells were then stained with an Annexin V-PE, 7-AAD Kit (BD, USA) for 15 min at room temperature in the dark. Flow cytometry was used to analyze cell apoptosis via a NovoCyte (Agilent, USA). Apoptotic cells (annexin V-PE-positive and 7-AAD-negative for early apoptosis and both annexin V-PE- and 7-AAD-positive for late apoptosis) were identified.

### Dual-luciferase reporter assay

The P1 and P2 sequences of the HIF1A-AS2 promoter were subsequently cloned and inserted into the luciferase reporter vector pGL3-basic for the promoter activity assay. The pGL3-P1, pGL3-P2, and pGL3-P2-MUT plasmids were transfected into 293T cells, and the luciferase activities were subsequently measured 48 h later. In addition, pGL3-P1, pGL3-P2,was co-transfected with pcDNA3.1-c-Jun or pGL3-basic, and a dual-luciferase reporter assay kit (Promega, USA) was used to measure luciferase activity after 48 h. Moreover, the HIF1A-AS2-WT, HIF1A-AS2-MUT, Radixin-WT and Radixin-MUT sequences were cloned and inserted into the luciferase reporter vector pmirGLO. HeLa cells were plated in 24-well plates, and then HIF1A-AS2-WT/MUT and Radixin-WT/MUT were co-transfected with miR-34b-5p mimics or miR-NC into HeLa cells with Lipo2000 reagent (Invitrogen, USA). Luciferase activity was subsequently measured 48 h later via a dual-luciferase reporter assay kit (Promega, USA). The experiments were conducted in triplicate and repeated three times.

### RNA immunoprecipitation assay

The binding between HIF1A-AS2 and miR-34b-5p, and between radixin and miR-34b-5p was explored by using an RNA-Binding Protein Immunoprecipitation Kit (EMD Millipore, USA) according to the manufacturer’s instructions. Briefly, the cells were lysed, and the resulting lysis solutions were subsequently incubated with an antibody against Ago2 (CST, 2897S, USA) or an isotype control IgG. The RNA-protein complexes were immunoprecipitated with protein A agarose beads, and the RNA-protein complexes were subsequently washed 5 times. After washing, the RNA/bead complexes were incubated with proteinase K at 55°C for 30 min to digest the proteins and purify the immunoprecipitated RNA. The final concentration of RNA was 115 ng/µL. The RNA was reverse transcribed to cDNA and analyzed by RT-qPCR. The relevant primers used are detailed in **Supplementary Table SVI**.

### Western blot assays

The cells were lysed with RIPA buffer (Thermo Fisher Scientific, USA) supplemented with protease inhibitors (CWBIO, Beijing, China). Of protein, 20µg was separated on 10% SDS-PAGE (Beyotime, Beijing, China) and then transferred to polyvinylidene difluoride (PVDF) membranes (Millipore, USA). The molecular weight of radixin is about 80 kDa, and in our study, the target protein band was between 70–100 kDa. The membranes were subsequently incubated overnight at 4°C with suitable dilutions of primary antibodies against the following antigens: radixin (1:2000; ab52495, Abcam, USA) and GAPDH (1:3000, ab9485, Abcam, USA). The membranes were subsequently washed with TBST three times for 10 min each in a shaker. After washing, the membranes were incubated with goat anti-rabbit secondary antibodies (1:5000, 7074P2, CST, USA) for 120 min at room temperature. The membranes were subsequently rewashed three times with TBST buffer. The protein bands were detected via enhanced chemiluminescence and imaged via a gel imager system (UE, UK). Finally, densitometric analysis of the protein bands was performed via ImageJ.

### Statistical analysis

SPSS Statistics 26.0 software (IBM, SPSS, Chicago, IL, USA) was used for analyzing the data. The measurement data were expressed as mean ± standard deviation. When data were normally distributed and had equal variance, t test was used for analyzing comparisons between two groups, while comparisons among multiple groups, one-way analysis of variance (ANOVA) with Tukey’s *post hoc* test. Paired samples were compared using a Wilcoxon matched pairs signed rank sum test. P<0.05 indicated statistically significant difference, while P<0.01 indicated extremely significant difference.

## Results

### Expression of HIF1A-AS2 and radixin are upregulated in CC tissues and cells

**Figure 1:**
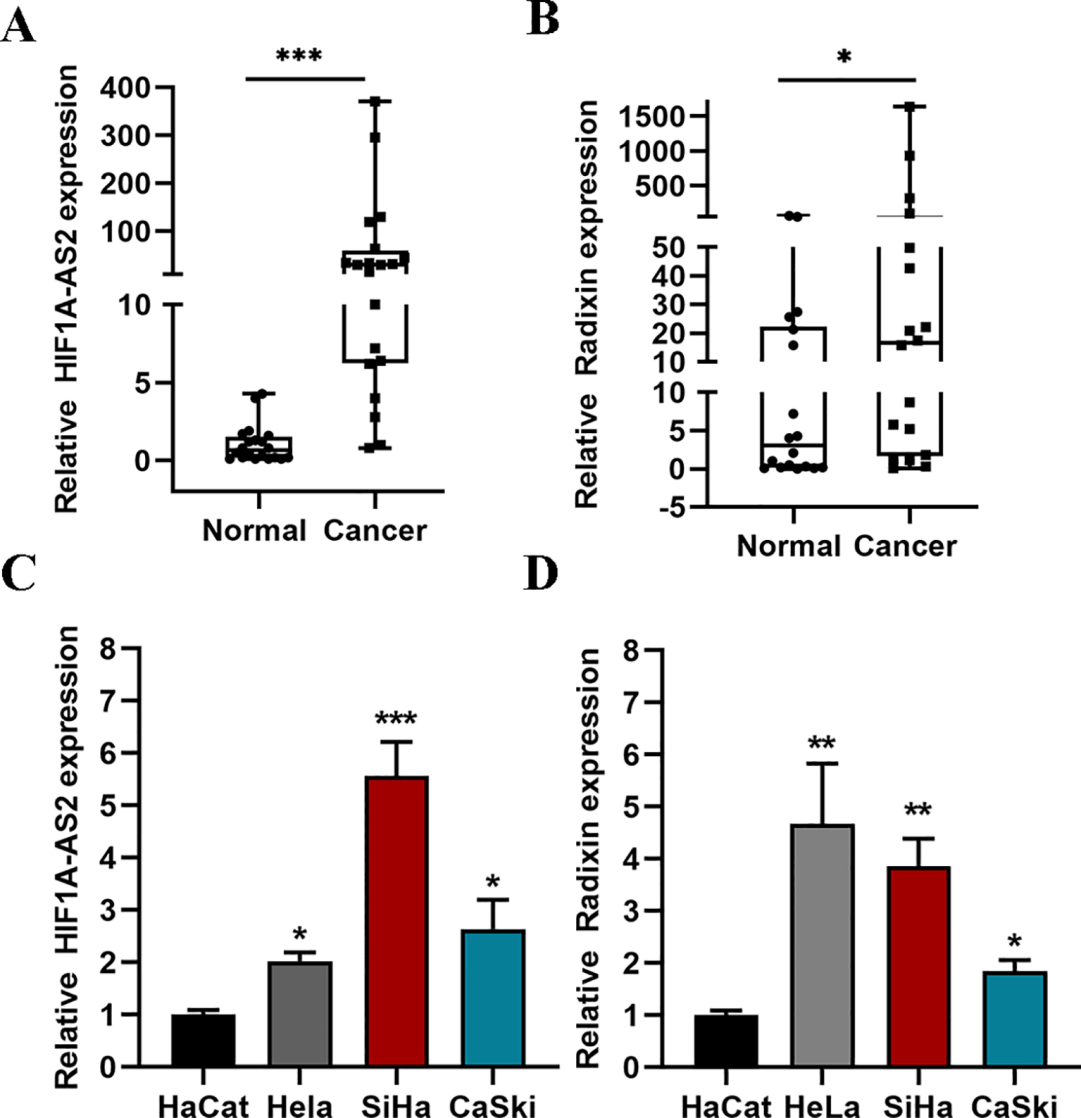
The expression of HIF1A-AS2 and Radixin. **(A, B)** The expression of HIF1A-AS2/Radixin mRNA in 20 cervical cancer samples and normal tissues was detected by qRT-PCR. Data were analyzed using the Wilcoxon Signed Rank Test. **(C)** The expression of HIF1A-AS2 in three CC cell lines (HeLa, SiHa, CaSki) and normal squamous epithelial cell (HaCat). **(D)** The expression of Radixin mRNA in three CC cell lines (HeLa, SiHa, CaSki) and normal squamous epithelial cell (HaCat). *P<0.05, ** P <0.01 and *** P <0.001.

To determine the expression of HIF1A-AS2 in CC tissues, we collected 20 cervical cancer patient tissues from 2020–2022. All biopsies of squamous type are shown in **Supplementary Table SV** 18 of 20 patients were diagnosed with squamous cell carcinomas and just 2 patients were diagnosed with adenocarcinomas. QRT-PCR was used to detect the expression of HIF1A-AS2 and radixin in 20 cervical cancer patient tissues and their matched normal tissue samples. The results revealed that the expression of HIF1A-AS2 was significantly greater in cervical cancer tissues (61.864 ± 100.309) than in normal tissues (1.054 ± 1.210) (P<0.01; **Figure 1A**). The expression of radixin (176.472 ± 42.6) was also greater than that in normal tissues (12.7 ± 19.76) (P<0.05; **Figure 1B**). To further explore the expression of HIF1A-AS2 in CC cells, the expression of HIF1A-AS2 and radixin was detected in CC cells via qRT-PCR, and the results revealed that HIF1A-AS2 was upregulated in three CC cell lines (HeLa (2.02 ± 0.19), Siha (5.56 ± 0.72) and Caski (2.63 ± 0.62)) compared with HaCat cells (1.0 ± 0.1) (P<0.05; **Figure 1C**). As for radixin, the results revealed that radixin was upregulated in three CC cell lines (HeLa (4.67 ± 1.15), Siha (3.85 ± 0.53) and Caski (1.84 ± 0.22)) compared with HaCat cells (1.0 ± 0.09) (P<0.05; **Figure 1D**).

### Knockdown of HIF1A-AS2 inhibits the proliferation and invasion and induced the apoptosis of CC cells

**Figure 2:**
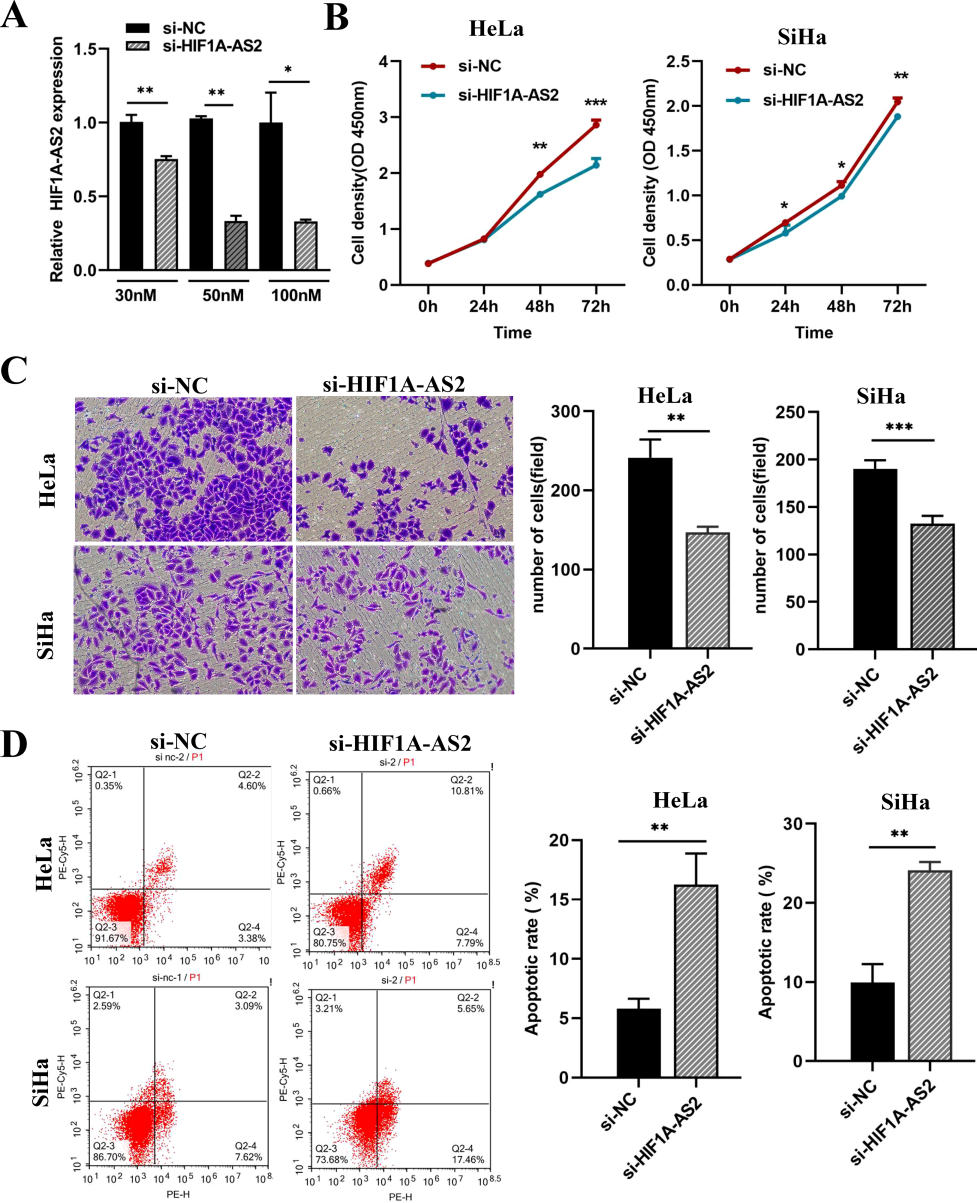
Knockdown of HIF1A-AS2 resulted in the suppression of proliferation and invasion, as well as the induction of apoptosis in cervical cancer cell lines. **(A)** The detection of knockdown efficiency for si-HIF1A-AS2. **(B)** The proliferation ability of HeLa and SiHa cells was determined by CCK-8 assays. The values are as follows, in Hela cells, 0h (si- NC 0.380 ± 0.005, si- HIF1A-AS2 0.391 ± 0.002), 24h (si- NC 0.825 ± 0.008, si- HIF1A-AS2 0.802 ± 0.007), 48h (si- NC 1.977 ± 0.017, si- HIF1A-AS2 1.623 ± 0.022), 72h (si- NC 2.862 ± 0.049, si- HIF1A-AS2 2.142 ± 0.067). In Siha cells, 0h (si- NC 0.288 ± 0.002, si- HIF1A-AS2 0.284 ± 0.001), 24h (si- NC 0.696 ± 0.017, si- HIF1A-AS2 0.580 ± 0.050), 48h (si- NC 1.114 ± 0.023, si- HIF1A-AS20.993 ± 0.014), 72h (si- NC 2.047 ± 0.025, si- HIF1A-AS2 1.882 ± 0.018). **(C)** The invasion ability of HeLa and SiHa cells was determined by transwell assays. The values are as follows, in Hela cells, si-NC 241.333 ± 13.295, si-HIF1A-AS2 147.000 ± 4.041. In Siha cells, si-NC190.400 ± 3.931, si-HIF1A-AS2 132.400 ± 3.749. **(D)** The apoptosis rate of HeLa and SiHa cells was detected by Flow Cytometer. The values are as follows, in Hela cells, si-NC 5.786 ± 0.490, si-HIF1A-AS2 16.253 ± 1.516. In Siha cells, si-NC 9.926 ± 1.342, si-HIF1A-AS2 24.123 ± 0.594. *P<0.05, ** P <0.01 and *** P <0.001.

The efficiency of siRNA knockdown was detected by qRT-PCR (P<0.01; **Figure 2A**), and 50 nM siRNA had the highest knockdown efficiency (si-HIF1A-AS2 0.346 ± 0.017, si-NC 1.000 ± 0.117) and was used for the following experiments. The results of the CCK-8 assay revealed that, compared with the control, the knockdown of HIF1A-AS2 inhibited the proliferation of both HeLa and SiHa cells (P<0.01; **Figure 2B**). Knockdown of HIF1A-AS2 significantly inhibited the invasion ability of both HeLa and SiHa cells (P<0.01; **Figure 2C**). To explore the cell apoptosis rate, flow cytometry was used, and the results demonstrated that knockdown of HIF1A-AS2 markedly increased the apoptosis rate of both HeLa and SiHa cells (P<0.01; **Figure 2D**).

### Overexpression of HIF1A-AS2 promoted the proliferation and invasion ability and impaired apoptosis of CC cells

**Figure 3:**
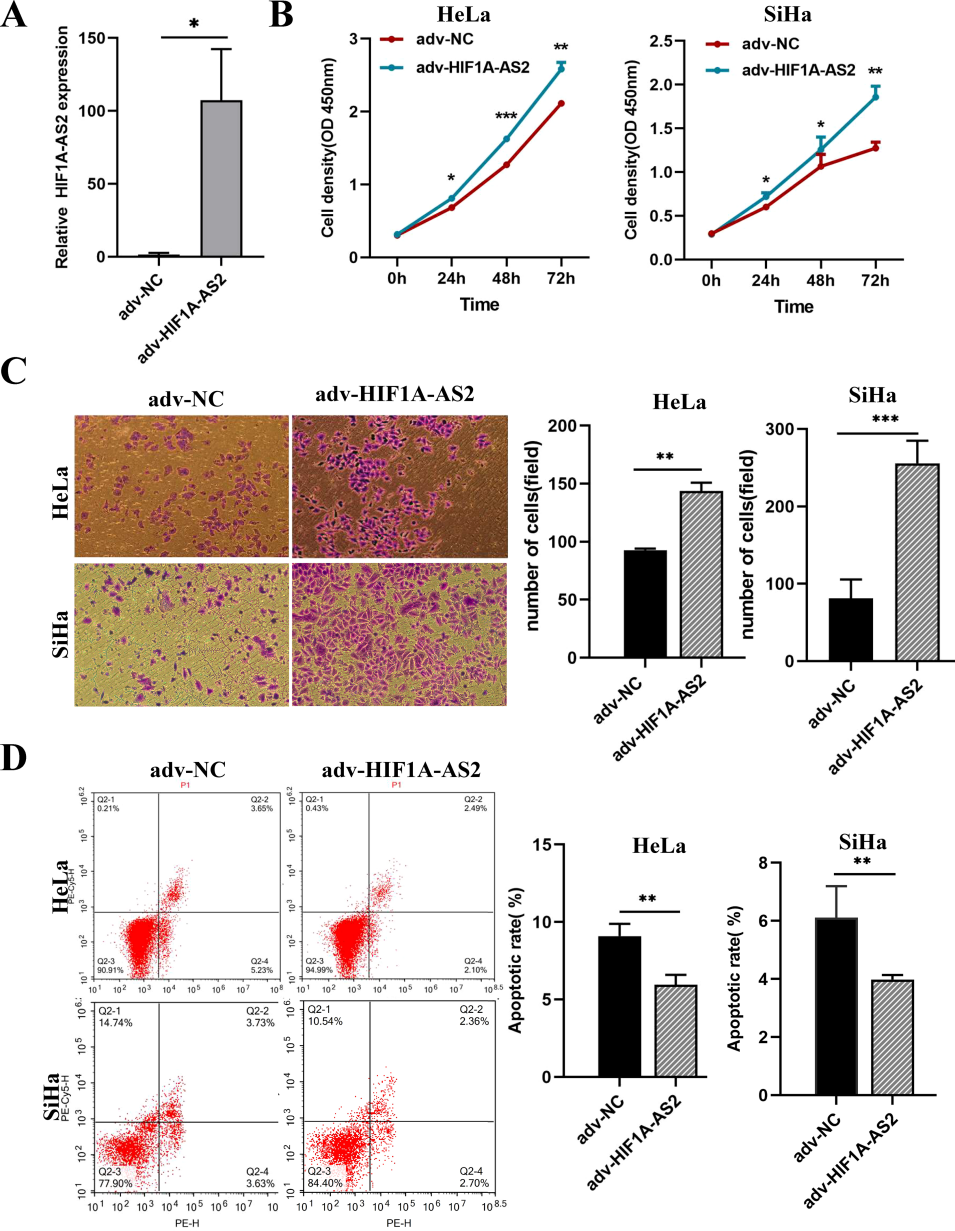
Overexpression of HIF1A-AS2 enhanced the proliferative and invasive capabilities in cervical cancer cells, while concurrently diminishing their rate of apoptosis. **(A)** The overexpression efficiency of Adenovirus (adv-HIF1A-AS2) was detected by qRT-PCR. **(B)** The proliferation ability of HeLa and SiHa cells was determined by CCK-8 assays. **(C)** The invasion ability of HeLa and SiHa cells was determined by transwell assays. **(D)** The apoptosis rate of HeLa and SiHa cells was detected by Flow Cytometer. *P<0.05, ** P <0.01 and *** P <0.001.

To elucidate the functions of HIF1A-AS2, the full-length cDNA of HIF1A-AS2 was cloned and inserted into an adenovirus encoding the green fluorescent protein (GFP) gene. The overexpression efficiency was determined using both fluorescence microscopy and qRT-PCR (P<0.05; **Figure 3A**). According to the results, an MOI = 50 was used for the following experiments.

The results of the CCK-8 assay revealed that overexpression of HIF1A-AS2 (adv-HIF1A-AS2) significantly promoted the proliferation of both HeLa and SiHa cells (P<0.01; **Figure 3B**). The Transwell assay results indicated that the invasion capacity of the HeLa and SiHa cells was significantly greater than that of the control cells (P<0.01; **Figure 3C**). Furthermore, the flow cytometry results revealed that, compared with the negative control (adv-NC), adv-HIF1A-AS2 impaired the apoptosis rate of both HeLa and SiHa cells (P<0.01; **Figure 3D**).

### c-Jun activates the expression of HIF1A-AS2 by binding to the HIF1A-AS2 promoter region

**Figure 4:**
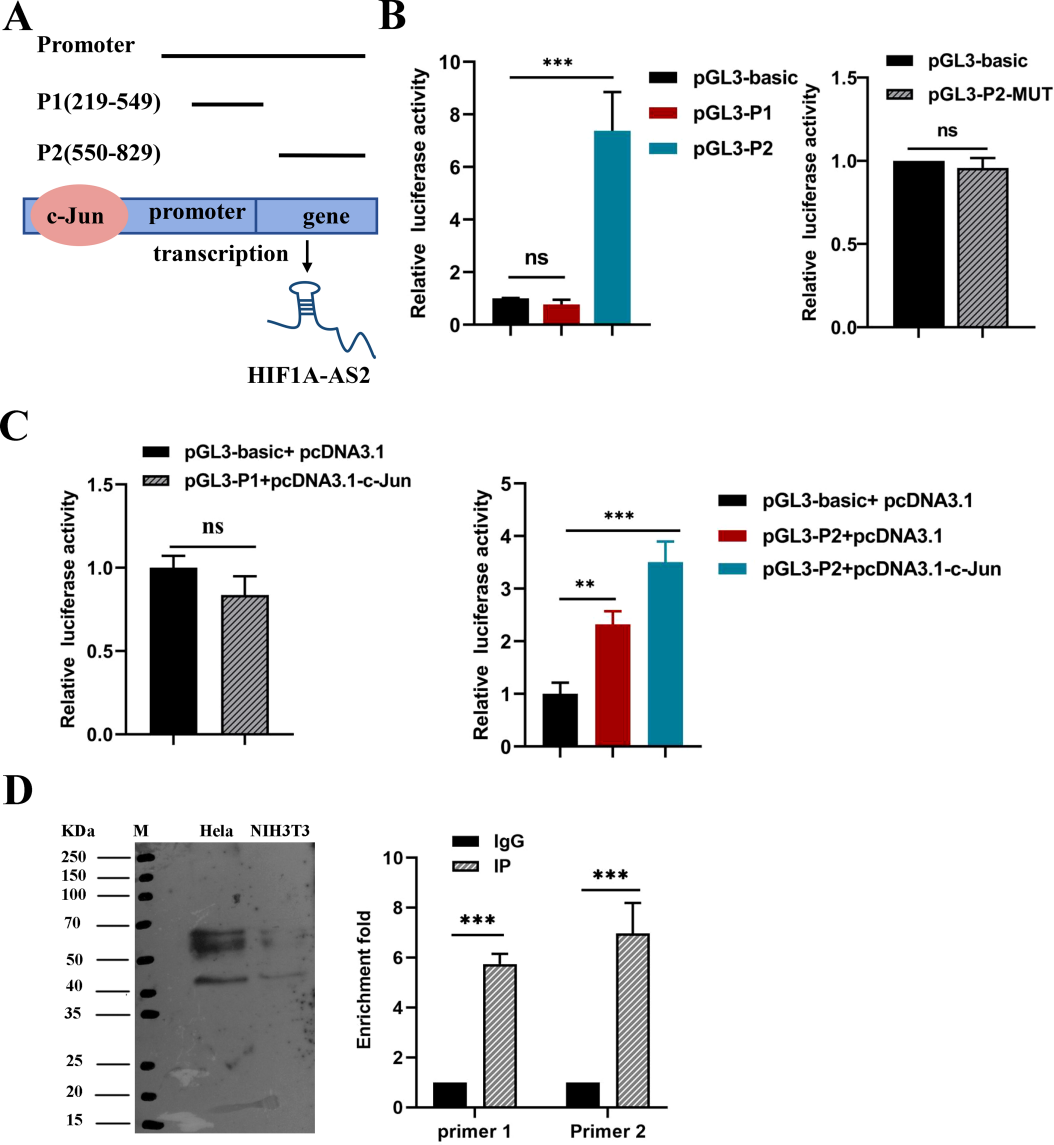
The transcription factor c-JUN caused high expression of HIF1A-AS2. **(A)** The two potential binding sites for c-Jun on the promoter region of HIF1A-AS2, named P1 and P2. **(B)** Promoter activity of P1, P2 sequences were analyzed by luciferase assay (Left); Luciferase activity had no significant change when mutated the binding sites of P2 sequence r compared to empty vector (Right). **(C)** Co-transfection of c-Jun overexpression plasmids,pcDNA3.1-c-Jun and pGL3-P1 couldn’t increase the relative luciferase activity(Left); Co-transfection of c-Jun overexpression plasmids,pcDNA3.1-c-Jun and pGL3-P2 could significantly increase the relative luciferase activity(Right).**(D)** ChIP assays were applied to validate the binding of c-JUN to the P2 sequence of the HIF1A-AS2 promoter. NIH3T3 was used as control cells which certainly expressed c-JUN. ** P <0.01 and *** P <0.001. ns means not significant.

The PROMO and JASPAR databases were used to predict the potential transcription factors of HIF1A-AS2 and binding sites, and all the predicted transcription factors are shown in **Supplementary Table SII**. These results indicate that c-Jun may be a crucial transcription factor. In addition, Liu C et al. reported that the expression of c-Jun was elevated in cervical intraepithelial neoplasia (19). Hence, c-Jun is considered a transcription factor that can bind to the promoter of HIF1A-AS2. According to the prediction data, there are two suggested binding sites between c-Jun and the promoter of HIF1A-AS2. To validate which binding sites had promoter activity, dual-luciferase assays were performed. We first constructed two dual-luciferase reporter vectors that contain two sequences of the HIF1A-AS2 promoter, including two binding sites, P1 and P2 (**Figure 4A**). The results revealed that luciferase activity increased when the pGL3-P2 vector was transfected, whereas pGL3-P1 had the same effect as the empty vector (P<0.01; **Figure 4B**). We subsequently constructed a pGL3-P2-MUT vector, and the results revealed that the luciferase activity did not significantly change compared with that of the empty vector (P>0.05; **Figure 4B** *)*. The above results indicate that the P2 sequence of the HIF1A-AS2 promoter has promoter activity.

To further explore whether HIF1A-AS2 was activated by c-Jun, pGL3-P2 and pcDNA3.1-c-Jun were cotransfected into 293T cells, and the results revealed that luciferase activity increased when pcDNA3.1-c-Jun and pGL3-P2 were cotransfected (P<0.01; **Figure 4C**). Moreover, ChIP assays confirmed that c-Jun could bind to the P2 sequence of the HIF1A-AS2 promoter (P<0.01; **Figure 4D**). Taken together, these results demonstrated that the expression of HIF1A-AS2 may be regulated by the transcription factor c-Jun in cervical cancer cells.

### miR-34b-5p is negatively regulated by HIF1A-AS2 in CC cells

**Figure 5:**
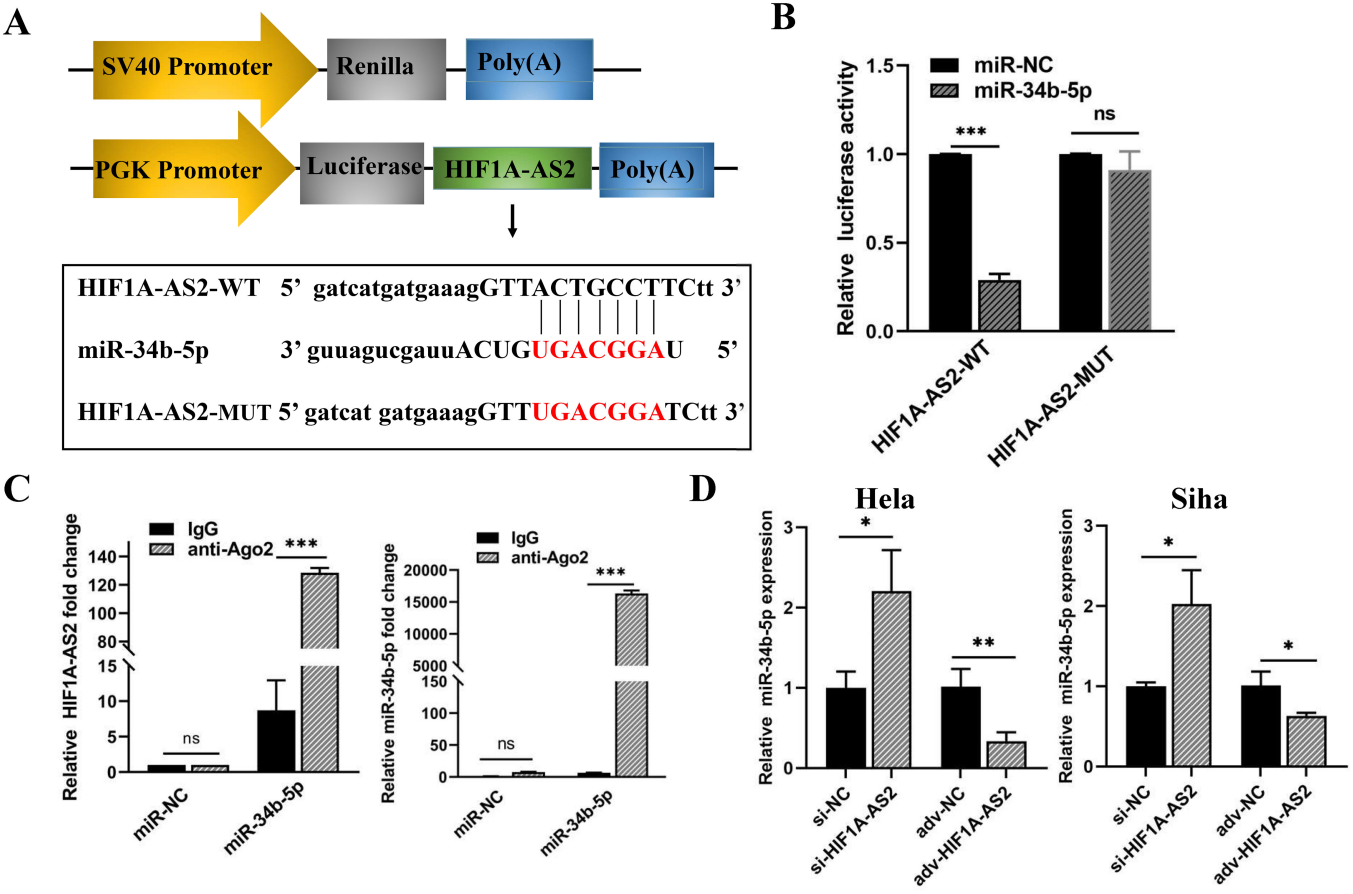
The miR-34b-5p was negatively regulated by HIF1A-AS2 in cervical cancer cells. **(A)** The predicted binding sites within HIF1A-AS2 and miR-34b-5p. **(B)** The binding relationship within HIF1A-AS2 and miR-34b-5p. **(C)** The interaction between HIF1A-AS2 and miR-34b-5p was verified in HeLa cells. **(D)** The relative expression of miR-34b-5p was detected by qRT-PCR after HIF1A-ARS2 overexpression or knockdown in HeLa and SiHa cells. *P<0.05, ** P <0.01 and *** P <0.001. ns means not significant.

The TargetScan and miRDB databases were used for the prediction of the target miRNAs of HIF1A-AS2 and the binding sites, and the prediction results are shown in **Supplementary Table SIII**. Research has reported that the expression of miR-34b-5p is reduced in cervical cancer and promotes the progression of cervical cancer (22). Therefore, miR-34b-5p was chosen, as shown in **Figure 5A**. miR-34b-5p has a binding site for HIF1A-AS2 (**Figure 5A**). To validate the interaction relationship, we constructed two sequences, one containing the binding site (HIF1A-AS2-WT) and the other containing the mutant binding site (HIF1A-AS2-Mut) (**Figure 5A**). These two sequences were cloned downstream of the luciferase reporter gene. The effects of miR-34b-5p on the HIF1A-AS2-WT or HIF1A-AS2-Mut luciferase reporter system were subsequently determined. The results demonstrated that cotransfection with wild-type (HIF1A-AS2-WT) plasmids and miR-34b-5p mimics significantly decreased the luciferase activity of HIF1A-AS2-WT but did not affect that of the mutant type (HIF1A-AS2-MUT) (P<0.01; **Figure 5B**).

The interaction of lncRNAs with miRNAs causes RNA-induced silencing complex (RISC) formation, and Ago2 is a critical protein of the RISC [12]. Therefore, we performed a RIP assay to further verify the binding interaction of anti-Ago2. qRT-PCR was used to determine the expression of HIF1A-AS2 and miR-34b-5p in the Ago2 immunoprecipitation complex. The results demonstrated that HIF1A-AS2 and miR-34b-5p were significantly enriched in Ago2-containing beads in both HeLa and Siha cells compared with those in the IgG group (*P*<0.01; **Figure 5C**).

To determine the regulatory relationship, we performed qRT-PCR to determine the expression of HIF1A-AS2 and miR-34b-5p in HeLa and Siha cells. The upregulation of HIF1A-AS2 led to a reduction in miR-34b-5p levels, whereas the silencing of HIF1A-AS2 resulted in an increase in miR-34b-5p expression, with statistically significant differences (*P*<0.01; **Figure 5D**). Taken together, these data revealed that HIF1A-AS2 could bind with miR-34b-5p and that the expression of miR-34b-5p was negatively regulated by HIF1A-AS2.

### HIF1A-AS2 regulated Radixin expression through sponging miR-34b-5p

**Figure 6:**
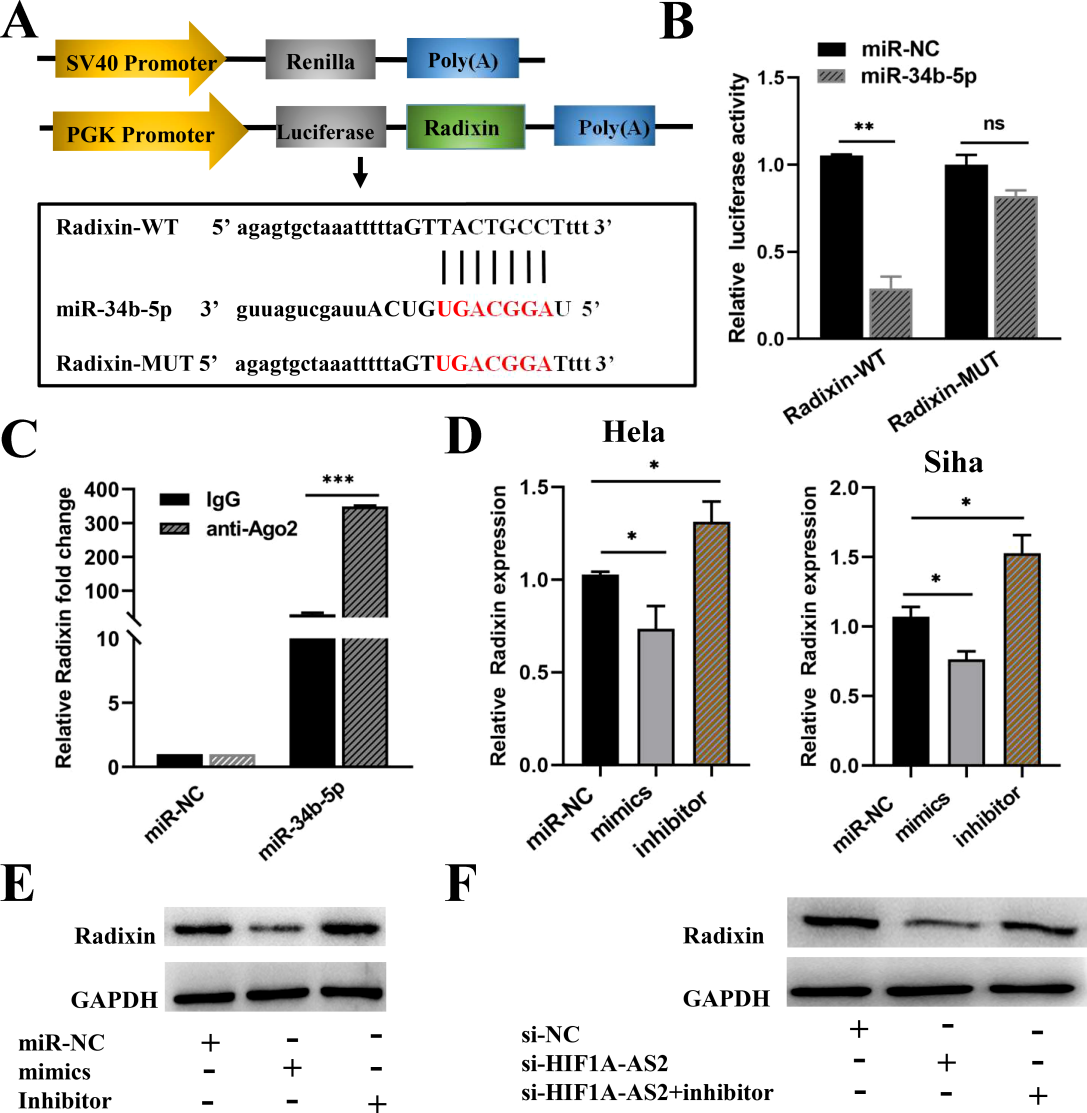
HIF1A-AS2 regulated radixin expression by sponging miR-34b-5p. **(A)** The binding sites between Radixin 3’UTR and miR-34b-5p were detected. **(B)** The binding relationship of Radixin and miR-34b-5p was determined. **(C)** RIP assays examined the enrichment of Radixin and miR-34b-5p Ago2-containing beads. **(D)** The relative expression of Radixin mRNA was detected after transfecting miR-34b-5p mimics, miR-34b-5p NC or miR-34b-5p inhibitors. **(E)** The protein expression of Radixin after transfecting miR-34b-5p mimics/miR-NC/miR-34b-5p inhibitors in Hela cells. **(F)** The relative protein expression of Radixin was determined by Western blot after transfecting si-HIF1A-AS2 or si-NC or si-HIF1A-AS2+miRNA inhibitors in Hela cells. *P<0.05, ** P <0.01 and *** P <0.001. ns means not significant.

miRNAs exert their functions by binding to their targets. TargetScan and miRDB were used to predict the targets of miR-34b-5p, and all the targets of miR-34b-5p are presented in **Supplementary Table SIV**. Specifically, increased expression of the radixin protein can promote the progression of cervical cancer (23). Therefore, radixin was considered a potential target, as shown in **Figure 6A**. To verify the interaction between radixin and miR-34b-5p, a dual-luciferase assay was performed as previously described. We constructed two sequences, one containing the binding site (Radixin-WT) and the other containing the mutant binding site (Radixin-Mut) (**Figure 6A**). These two sequences were cloned downstream of the luciferase reporter gene. The effects of miR-34b-5p on the Radixin-WT or Radixin-Mut luciferase reporter system were subsequently determined. The results revealed that the luciferase activity decreased when the cells were cotransfected with wild-type (Radixin-WT) and miR-34b-5p mimics but had no significant effect on the mutant type (Radixin-MUT) (*P*<0.01; **Figure 6B**). The results of the luciferase activity assay indicated that miR-34b-5p might bind to radixin mRNA.

Moreover, to confirm that miR-34b-5p could indeed bind with radixin mRNA in CC cells, a RIP assay was performed using anti-Ago2. The results revealed that the expression of both miR-34b-5p and radixin mRNAs was increased in the anti-Ago2 immunoprecipitation complex (*P*<0.001; **Figure 6C**). Subsequently, qRT-PCR was used to explore how miR-34b-5p regulates the expression of radixin mRNA. We transfected miR-34b-5p mimics to overexpress miR-34b-5p and miR-34b-5p inhibitors to knockdown miR-34b-5p in both HeLa and Siha cells, the inhibiting effect of miR-34b-5p was shown in **Supplementary Figure S1**. The results revealed that the overexpression of miR-34b-5p decreased the expression of radixin mRNA, whereas the knockdown of miR-34b-5p increased the expression of radixin mRNA (*P*<0.05; **Figure 6D**). In addition, we performed Western blot experiments to further verify the regulatory relationship. Compared with the control, transfection with the miR-34b-5p mimics decreased the amount of the radixin protein, whereas the miR-34b-5p inhibitors increased the amount of the radixin protein (**Figure 6E**, **Supplementary Figure S2**-**Supplementary Figure S5**). The above results confirmed that miR-34b-5p could negatively regulate the expression of radixin at both the mRNA and protein levels. In addition, western blot results revealed that the expression of the radixin protein decreased when cells were transfected with si-HIF1A-AS2 to knockdown HIF1A-AS2, whereas the expression of the radixin protein increased when they were cotransfected with si-HIF1A-AS2 and miR-34b-5p inhibitors (**Figure 6F**). These findings indicated that HIF1A-AS2 could bind with miR-34b-5p to positively regulate radixin.

## Discussion

Despite the application of the HPV vaccine, the mortality and morbidity rates of CC remain high (4). In this study, we revealed that HIF1A-AS2 was highly expressed in the tissues of 20 CC patients and three different cell lines. Subsequent experiments revealed that knockdown of HIF1A-AS2 suppressed proliferation and invasion and induced apoptosis in HeLa and Siha cells. Conversely, the overexpression of HIF1A-AS2 enhanced these cellular processes. Furthermore, we investigated the mechanisms underlying the upregulation of HIF1A-AS2 in CC cells. These results indicate that the nuclear transcription factor c-Jun can regulate HIF1A-AS2. Additionally, guided by the ceRNA hypothesis and bioinformatics predictions, we postulated that the HIF1A-AS2/miR-34b-5p/Radixin axis might play a crucial role in this molecular mechanism. Our experimental data corroborated this hypothesis, demonstrating that HIF1A-AS2 can act as a molecular sponge for miR-34b-5p, thereby modulating the expression of radixin.

HIF1A-AS2 has been reported to be a potential oncogene in various cancer types, including gastric cancer (12), breast cancer (14) and bladder cancer (15). Guo et al. (14) reported that HIF1A-AS2 was highly expressed in breast cancer and that the knockdown of HIFIA-AS2 suppressed the metastasis of breast cancer cells. Chen et al. (16) reported that a high level of HIF1A-AS2 was associated with advanced stages of renal carcinoma. In addition, HIF1A-AS2 is involved in the progression of other diseases, such as angiogenesis (24), osteogenic differentiation of adipose stem cells (25), atherosclerosis (26), and preeclampsia (27). However, in CC, the expression and functions of HIF1A-AS2 are still poorly understood. Our research revealed that HIF1A-AS2 expression was elevated in 20 CC tissue specimens and across three distinct cell lines compared with the normal tissues. There is one study which published in 2022 investigated that HIF1A-AS2 was significantly increased in HPV 16, HPV 18 positive cervical cancer tissue, and in the HPV- positive cervical cancer cells (28). Our results are consistent with this study. But the mechanism is totally different. One limitation of our study is that the number of tissues used is relatively small, only 20 tissue samples were used. In addition, sample heterogeneity and subgroup differences could’t be excluded. Thus, more tissue samples will be collected in the near future and verify the possibility of HIF1A-AS2 as a prognostic molecular marker for cervical cancer.

In most previous studies, HIF1A-AS2 was found to promote cancer cell proliferation, invasion, and migration and inhibit cell apoptosis. This study found that manipulations involving either the knockdown or overexpression of HIF1A-AS2 affected the proliferation, invasion, and apoptosis of CC cells. These findings imply that HIF1A-AS2 may play a tumorigenic role in the progression of cervical cancer. Besides, it’s known that HIF1A-AS2 is an antisense transcript which could bind to the 3’ untranslated region (3’uTR) of Hypoxia Inducible Factors 1α in 1999 (11).Hypoxia is a hallmark of rapidly growing tissues such as developing embryos and solid tumors. The cellular response to hypoxic stress involves the activation of Hypoxia Inducible Factors (HIFs), a group of DNA-binding proteins that induce transcription of numerous genes involved in angiogenesis, glycolysis, metabolic adaptation, erythropoiesis and cell survival (29). Some studies have shown that the expression of HIF1A-AS2 was upregulated under hypoxia (30–32). Tsai-Tsen Liao et al. showed that HIF1A-AS2 was upregulated in hypoxic tumor cells and hypoxic tumor-derived exosomes in head and neck squamous cell carcinoma (HNSCC). Hypoxia-inducible factor 1 alpha (HIF1α) was found to directly bind to the regulatory region of HIF1A-AS2 to enhance its expression. Ultimately, increased HIF1A-AS2 promoted immune evasion of cancer cells (30). Ewida, Heba A et al. showed that remarkable upregulation of lncRNA HIF1A-AS2 and HIF1-α was noticed in all stroke groups relative to controls. And angiogenesis was activated (32).Our further studies will be conducted to explore if HIF1A-AS2 promote proliferation of CC cells under hypoxia.

Dysregulated transcription factors (TFs) can promote tumorigenesis by transcriptionally regulating noncoding RNAs, such as circular RNAs (circRNAs) and long noncoding RNAs (lncRNAs) (33). Transcription factors can specifically recognize lncRNA promoter sequences, thereby promoting their expression (34). For example, Wang et al. (35) reported that the transcription factor USF1 activates HAS2-AS1 transcription by recognizing and binding the lncRNA HAS2-AS1 promoter region, resulting in increased invasion and migration of glioma cells. Therefore, we hypothesized that the elevated expression of HIF1A-AS2 could be attributed to the influence of a pivotal transcription factor. Through bioinformatics prediction, we found that c-Jun may be a crucial regulator of HIF1A-AS2. Besides, C-Jun has been demonstrated to be elevated in many types of cancer and is involved in tumorigenesis (21, 36). Interestingly, the expression of c-Jun has been reported to be increased in CC tissues (19). This means c-Jun may regulate the expression of HIF1A-S2 by binding with the promoter of HIF1A-AS2. We did ChIP and luciferase reporter assays, and the results confirmed that c-Jun actively interacts with the promoter region of HIF1A-AS2, thereby increasing the transcription of the HIF1A-AS2 gene. In addition,

Studies have reported c-JUN interacts with various signaling pathways, included EGFR-ERK, EGFR-RhoA-ROCK, activin B-MAP3K1-JNK and etc (37). Further studies can be conducted to identify the specific factors or signaling pathways involved in CC.

To date, the most studied aspect of lncRNA function is as a sponge for microRNAs (miRNAs), which means that lncRNAs can competitively bind to miRNAs and reduce the binding between miRNAs and their target mRNAs, increasing the expression of their target genes (38). MiRNAs are noncoding RNAs that act as oncogenes of tumor suppressor genes and are involved in the tumorigenesis of solid tumors and the progression of other diseases (39). Bioinformatics analysis revealed that miR-34b-5p may be a crucial target of HIF1A-AS2. In addition, downregulated miR-34b-5p

can inhibit cervical cancer proliferation and invasion (22). Our conjecture is consistent with a previous report. We confirmed that HIF1A-AS2 directly binds to miR-34b-5p by performed RIP and luciferase reporter assays, and a qRT-PCR assay demonstrated that miR-34b-5p was negatively regulated by HIF1A-AS2. MiRNAs are capable of degrading or inhibiting the transcription of target genes through their specific binding to the 3′-untranslated regions (3′-UTRs) of the corresponding mRNAs. Through bioinformatics analysis, we subsequently revealed that radixin is a target gene of miR-34b-5p. It was demonstrated that miR-34b-5p may interact with the 3’UTR of radixin via RIP and luciferase reporter assays and qRT-PCR assays, which revealed that radixin mRNAs are negatively regulated by radixin mRNAs. Moreover, we confirmed that miR-34b-5p negatively regulated the expression of radixin at the protein level. In addition, this study verified that HIF1A-AS2 positively regulated the expression of radixin at the protein level. These results are consistent with the ceRNA hypothesis. In addition, deeper molecular mechanism of radixin could strengthen the depth of our research, for example, which signaling pathway was activated thereby promoted CC cells proliferation, invasion and inhibited apoptosis. We will consider it seriously in our post-study.

At present, cancer treatment has entered the era of targeted therapy. RNA therapeutics (RNATx) has been gaining significant momentum (40). RNATx aim to treat diseases, including cancer, by targeting or employing RNA molecules for therapeutic purposes. LncRNAs is the most promising targets which regulate oncogenic molecular networks in a cell type-restricted manner (41). At present, therapies based on lncRNA include Antisense oligonucleotides (ASO), Small interfering RNAs (SiRNA), Crispr-Cas and small molecule compound (42). These therapies are mainly achieved by reducing the expression of lncRNA or altering the structure of lncRNA. Despite achieving success in treating certain tumors, there still remain some limitations. A broader challenge for RNATx is successfully delivering large, unstable and negatively charged molecules into the correct subcellular location of a target cell type, while avoiding toxic accumulation and activation of innate immune responses (41). At present, RNA therapy targeting HIF1A-AS2 is very rare, but as technology advances, it is promising.

In summary, a tumor-related lncRNA, HIF1A-AS2, was identified, which is frequently overexpressed and associated with CC progression. For molecular mechanisms, most studies have focused on downstream molecular mechanisms, while in our research, the upstream molecular mechanism has also been explored. The results showed that HIF1A-AS2 was activated by the transcription factor c-Jun and acted as a sponge for miR-34b-5p to inhibit the radixin gene. Our findings suggest that HIF1A-AS2 may be a viable and promising therapeutic target and biomarker for CC.

## Conclusions

The results demonstrated that HIF1A-AS2 is overexpressed in cervical cancer. c-Jun was shown to interact with the promoter region of HIF1A-AS2, thereby facilitating the transcription of the HIF1A-AS2 gene. Additionally, HIF1A-AS2 serves as a molecular sponge for miR-34b-5p, regulating radixin expression, which contributes to the advancement of malignant traits in cervical cancer cells. These observations indicate that HIF1A-AS2 plays an oncogenic role in the development of cervical cancer. In conclusion, the interactions within the HIF1A-AS2-miR-34b-5p-RDX axis are crucial for cervical cancer progression, highlighting HIF1A-AS2 and miR-34b-5p as promising biomarkers for cancer diagnosis and prognosis.

## Data Availability

The original contributions presented in the study are included in the article/**Supplementary Material**. Further inquiries can be directed to the corresponding author.

## Supplementary material

Supplementary Figure 1 The inhibiting effect of miR-34b-5p by transfecting miR-34b-5p inhibitors. *P<0.05, ** P <0.01 and *** P <0.001.

Supplementary Figure 2 The protein levels of radixin were measured by western blot. The protein expression of radixin after transfecting miR-34b-5p mimics/miR-NC/miR-34b-5p inhibitors(concentration:30nM,50nM).

Supplementary Figure 3 The relative protein expression of radixin was determined by Western blot after transfecting si-HIF1A-AS2 or si-NC or si-HIF1A-AS2+miRNA inhibitors(concentration:30nM,50nM).

Supplementary Figure 4 The protein expression of radixin after transfecting miR-34b-5p mimics/miR-NC/miR-34b-5p inhibitors(concentration:50nM).

Supplementary Figure 5 The relative protein expression of radixin was determined by Western blot after transfecting si-HIF1A-AS2 or si-NC or si-HIF1A-AS2+miRNA inhibitors (concentration:50nM). **Supplementary Figure S2** and **Supplementary Figure S3** were the first repeated experiment, **Supplementary Figure S4** and **Supplementary Figure S5** were the second repeated experiment. si-NC, siRNA negative control; mimics, miR-34b-5p; inhibitors, miR-34b-5p inhibitors; si-HIF1A, siRNA for HIF1A-AS2)
