# Supplementary figures and images for: lncRNA HIF1A-AS2 acts as an oncogene to regulate malignant phenotypes in cervical cancer
Source: Front Oncol. 2025 Feb 27;15:1530677. doi: 10.3389/fonc.2025.1530677 (PMC11912943; doi:10.3389/fonc.2025.1530677)

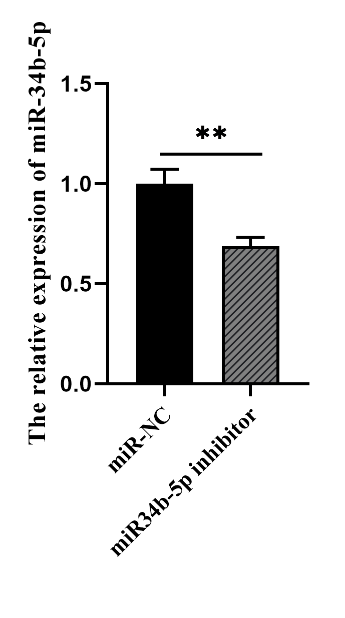

Supplement: Supplementary Figure 1 — The inhibiting effect of miR-34b-5p by transfecting miR-34b-5p inhibitors. *P<0.05, ** P <0.01 and *** P <0.001. [file Image1.tif]

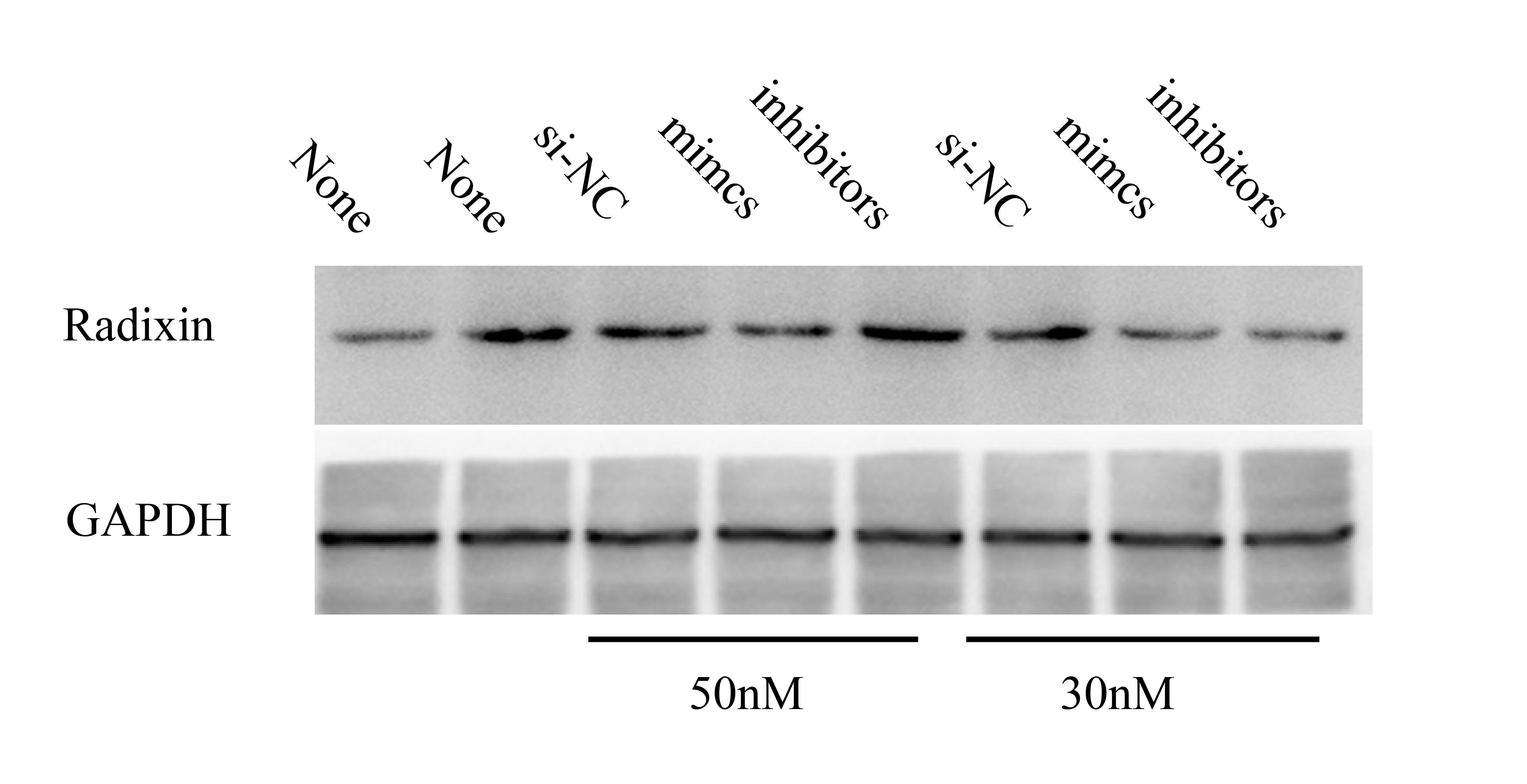

Supplement: Supplementary Figure 2 — The protein levels of radixin were measured by western blot. The protein expression of radixin after transfecting miR-34b-5p mimics/miR-NC/miR-34b-5p inhibitors(concentration:30nM,50nM). [file Image2.tif]

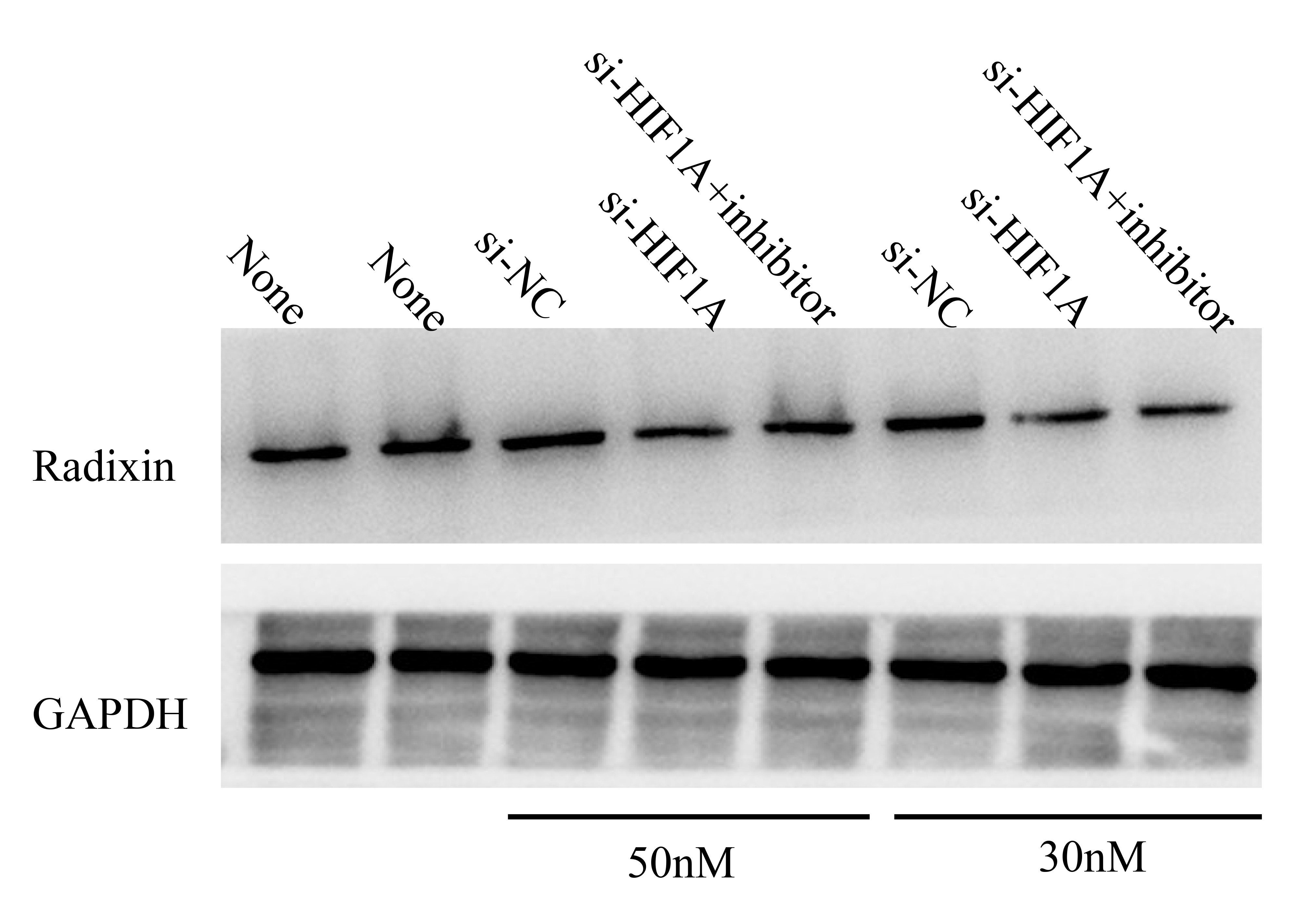

Supplement: Supplementary Figure 3 — The relative protein expression of radixin was determined by Western blot after transfecting si-HIF1A-AS2 or si-NC or si-HIF1A-AS2+miRNA inhibitors(concentration:30nM,50nM). [file Image3.tif]

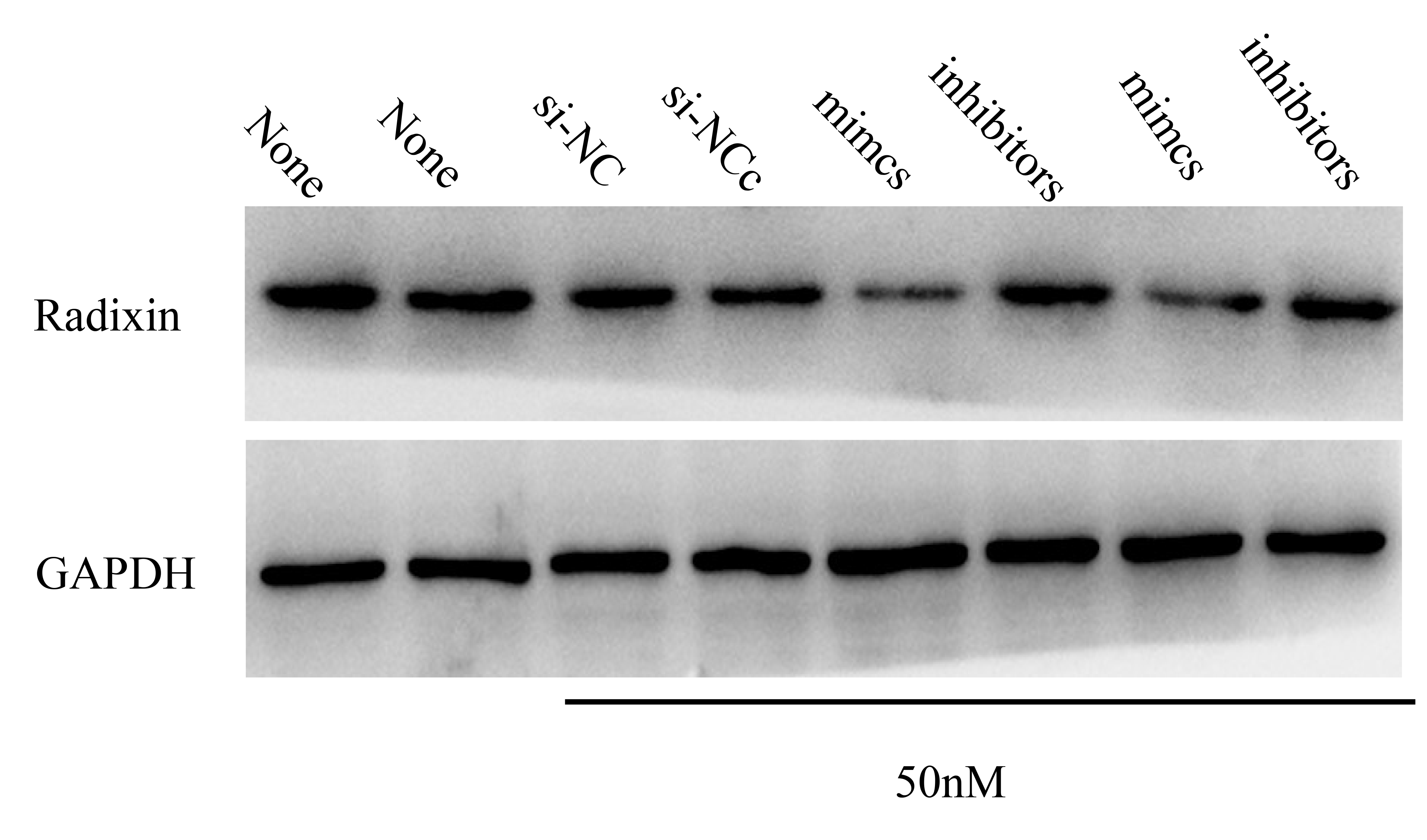

Supplement: Supplementary Figure 4 — The protein expression of radixin after transfecting miR-34b-5p mimics/miR-NC/miR-34b-5p inhibitors(concentration:50nM). [file Image4.tif]

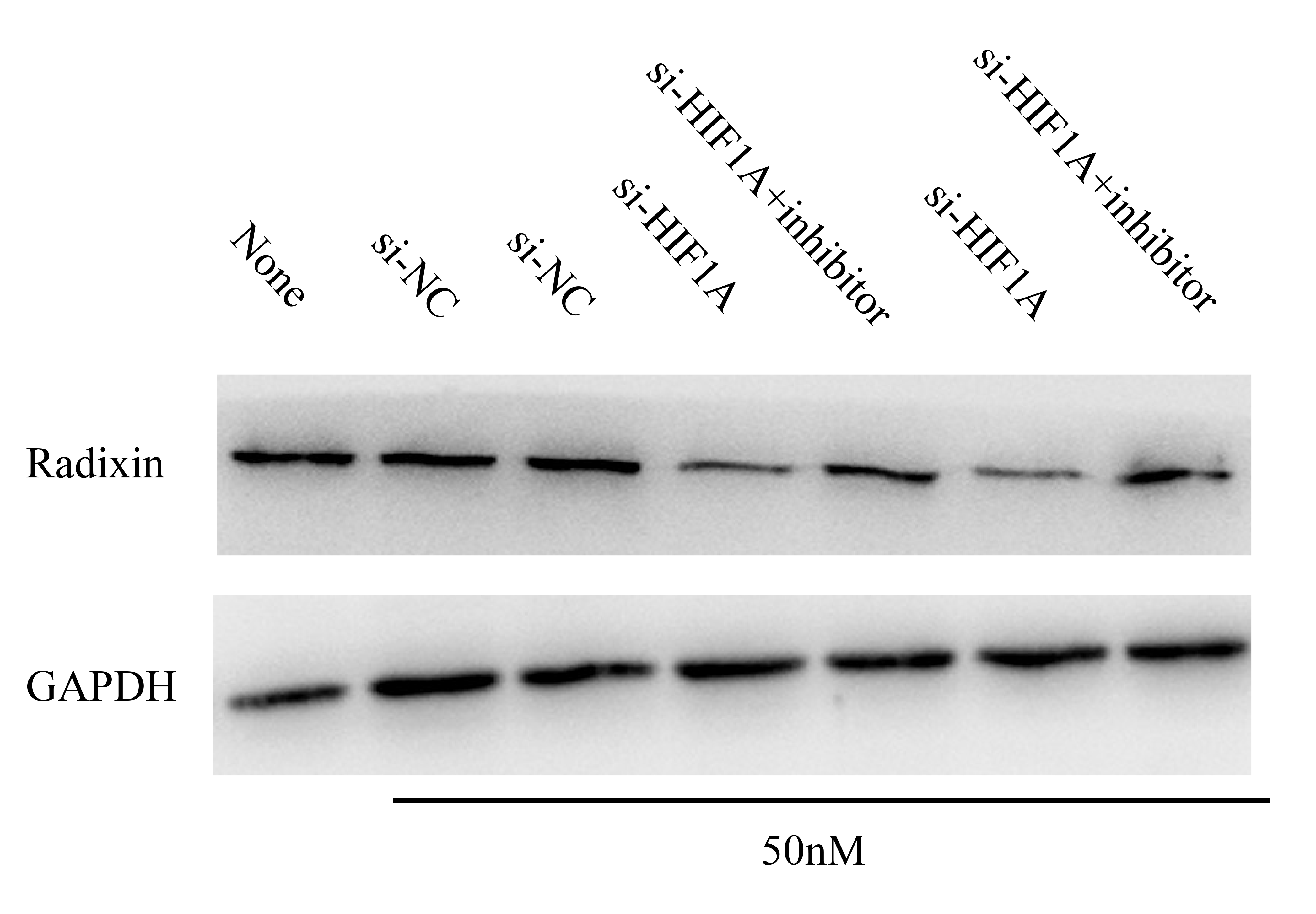

Supplement: Supplementary Figure 5 — The relative protein expression of radixin was determined by Western blot after transfecting si-HIF1A-AS2 or si-NC or si-HIF1A-AS2+miRNA inhibitors (concentration:50nM). Supplementary Figure S2 and Supplementary Figure S3 were the first repeated experiment, Supplementary Figure S4 and Supplementary Figure S5 were the second repeated experiment. si-NC, siRNA negative control; mimics, miR-34b-5p; inhibitors, miR-34b-5p inhibitors; si-HIF1A, siRNA for HIF1A-AS2) [file Image5.tif]
