# Supplementary material for: lncRNA HIF1A-AS2 acts as an oncogene to regulate malignant phenotypes in cervical cancer
Source: Front Oncol. 2025 Feb 27;15:1530677. doi: 10.3389/fonc.2025.1530677 (PMC11912943; doi:10.3389/fonc.2025.1530677)
Supplement: Supplementary file 6 [file Table1.docx]

Table SI. The promotor sequence of HIF1A-AS2.

| **Name** | **Sequence** | |
| --- | --- | --- |
| Promoter sequence of HIF1A-AS2 | | TCCTTTTGTTCAGTATATATACCTCAGGGTAAAGGACCTAAGGCTCTGGCACTTCCTACATAATTTGTTCATAAAAATAATTTTCTGTACCTGTAGGTGCAGGGATTCATAGTGATTTTTCTGTTTTAAAAAATATCTTTCTACACTGTCTGATTTTTAAAAATGAGATTATCAGTATAAAAGAAATACAGCTATTTTGTAAAAGAAAAATACAGGAGAAAAAGGATAAAGCTACTTTGTTTTAAAACGCAGTATCATTAAAACAAAAACAAACCAAATTTGTTCTAAGTTTGACTTTAGAGTCAGGAAACTTAAGCTTACATTTTTGGTCTGCCATCTATTACTTTTAAAGCTTGGGCAAATTATTCATTTGAAGTCTAAATTTATTAATCTGTTAATGGGAACAGATTAGAAATCTTCAGAGAAGCTCTAGCCTTTGTAACATTGTGACTATAATGCTGAGAACTGCTTCACTCATCCCATTCATATTTTAAAAATACTAATATTTTGTGTTTGAGCATTTTAATAGGCTCAGAAACTTAAAAATGATGTTTCTTTTCTAACAACATACTCTTTTCAATGGGATATTATGGTTGTTATTATTTAACATGACATTTAGGGACTCAACATACATTAAGGTGATGGCACTAAGATAAATGTAGAAATAACCAGTACCATGTAATTTTCATAAGTGCTTAAATTGTTGGTAAACAATTTTATGAGTTGGGGTGTTGAAGCAAATATTATTAATATTTGAACATAAAAGCTGATCAAAGGGGCCTGGTCCACAGAAGATGTTTATTTGATGTAACAAAACAATACAGTTAGTGTTAGATCCAACCACAAAGAGCAAAAGGAATGAAAAATTTGTACAATGTACATAGAAAAAACAAGATATTTACTGTGACAACTATATATTCCTAAAATAATGCTTCTAAAATTACTCAATTATTGAAATCTACATGAAAAAAAGGATGTTAATAGCGACAAAGTGCATAAAATCAAACATTGTATTTTGAGCAAATTAACATACTAGGCAATTTTGCTAAGAATGCATGATTTTTTTTTTCTTGTTTACAGTCTGCTCAAAATATCTTTATACCAACAGGGTAGGCAGAACATTTAGGTTTAATATCAGTTACACAATATT  AGCATAAACTTCCACAACTACATAGGGTATTGTTTTCTTTTGAGCTGGCAAAGTGACTATAGAAACATCAGATGATTTCTCTGAATTGAGAATTTTATCCAAATAAATGCCACATACCTTCTAGATATATGCATATCTTTCTATATTATGTAAATGGCTTTACCCATTTAAATAATAAACCATACAGCATTTAAGAATCATTATTATATGATTAACAATGTCATGTTCCAGGTTTAACAATTTCATAGGCCAAAAAAAATTTCTTCTTAAAAACTAGTTTTATAAACGCAGAATATATTCCATGAGTAACTGCTGGTATTTTTTAAGAAAATATATTGTGCAATTGTGGCTACCACGTACTGCTGGCAAAGCATTATTATTTATGTAAAATGTGAAAAAAAAGGTGTAAAAATTTTTCAACTGCCTATGATCATGATGAAAGGTTACTGCCTTCTTACAAAAATTATATTGGCATCTTCTTAAAAATAATTCGAAAAAGGGATAAACTCCCTAGCCAAAAATAAATAAATAAAAAGGTGCATTTTTTAAAATGATGCTACTGCAATGCAATGGTTTAAATACCAAAAAACTGAGAAAATGAGCTGTCTGTGATCCAGCATTAAAGAACATACTAAAAAAGAGCATTAATGTAAATTAAGTAGAAAGGGGATCAAAATTGAACTAACCAAGTTTGTGCAGTATTGTAGCCAGGCTTCTAAAATTAGATGTAGAAAATATAAATAGACTGCTTTAGGTAATGAGCCACCAGTGTCCAAAAAAAGGAATGAAATTAAGAAAAAGCTCAGTTAACTTGATCCAAAGCTCTGAGTAATTCTTCACCCTGCAGTA |
