# Supplementary material for: lncRNA HIF1A-AS2 acts as an oncogene to regulate malignant phenotypes in cervical cancer
Source: Front Oncol. 2025 Feb 27;15:1530677. doi: 10.3389/fonc.2025.1530677 (PMC11912943; doi:10.3389/fonc.2025.1530677)
Supplement: Supplementary file 7 [file Table2.docx]

Table SII. The 40 predicted transcription factors for HIF1A-AS2.

| Rank | NAME | MATRIX_WIDTH |
| --- | --- | --- |
| 1 | C/EBPbeta [T00581] | 4 |
| 2 | GR-alpha [T00337] | 5 |
| 3 | EBF [T05427]; 11 | 11 |
| 4 | AP-2alphaA [T00035] | 6 |
| 5 | ENKTF-1 [T00255] | 8 |
| 6 | TFII-I [T00824] | 6 |
| 7 | STAT4 [T01577] | 6 |
| 8 | c-Ets-1 [T00112] | 7 |
| 9 | Elk-1 [T00250] | 9 |
| 10 | ELF-1 [T01113] | 13 |
| 11 | PU.1 [T02068] | 13 |
| 12 | GR-beta [T01920] | 5 |
| 13 | TFIID [T00820] | 7 |
| 14 | MEF-2A [T01005] | 11 |
| 15 | HNF-3alpha [T02512] | 8 |
| 16 | GATA-1 [T00306] | 6 |
| 17 | XBP-1 [T00902] | 6 |
| 18 | TBP [T00794] | 10 |
| 19 | STAT5A [T04683] | 13 |
| 20 | LEF-1 [T02905] | 8 |
| 21 | FOXP3 [T04280] | 6 |
| 22 | GATA-2 [T00308] | 9 |
| 23 | PR B [T00696] | 7 |
| 24 | PR A [T01661] | 7 |
| 25 | GR [T05076] | 7 |
| 26 | NFI/CTF [T00094] | 8 |
| 27 | ATF3 [T01313] | 8 |
| 28 | AP-1 [T00029] | 9 |
| **29** | **c-Jun [T00133]** | **7** |
| 30 | c-Fos [T00123] | 10 |
| 31 | c-Ets-2 [T00113] | 9 |
| 32 | IRF-1 [T00423] | 9 |
| 33 | NF-AT1 [T00550] | 9 |
| 34 | c-Myb [T00137] | 8 |
| 35 | NF-1 [T00539] | 8 |
| 36 | YY1 [T00915] | 4 |
| 37 | HNF-1C [T01951] | 9 |
| 38 | HNF-1B [T01950] | 9 |
| 39 | C/EBPalpha [T00105] | 7 |
| 40 | PEA3 [T00685] | 9 |

There are over 200 predicted transcription factors. Due to the large amount data, the table shows only 40 data. The ranking is not based on prediction scores.
