# Supplementary material for: lncRNA HIF1A-AS2 acts as an oncogene to regulate malignant phenotypes in cervical cancer
Source: Front Oncol. 2025 Feb 27;15:1530677. doi: 10.3389/fonc.2025.1530677 (PMC11912943; doi:10.3389/fonc.2025.1530677)
Supplement: Supplementary file 8 [file Table3.docx]

Table SIII. All predicted miRNAs for HIF1A-AS2.

| Target Rank | Target Score | miRNA Name | Gene Symbol |
| --- | --- | --- | --- |
| 1 | 93 | [hsa-miR-153-5p](https://mirdb.org/cgi-bin/mature_mir.cgi?name=hsa-miR-153-5p) | submission |
| 2 | 92 | [hsa-miR-4724-5p](https://mirdb.org/cgi-bin/mature_mir.cgi?name=hsa-miR-4724-5p) | submission |
| 3 | 92 | [hsa-miR-548n](https://mirdb.org/cgi-bin/mature_mir.cgi?name=hsa-miR-548n) | submission |
| 4 | 91 | [hsa-miR-6751-3p](https://mirdb.org/cgi-bin/mature_mir.cgi?name=hsa-miR-6751-3p) | submission |
| 5 | 87 | [hsa-miR-155-5p](https://mirdb.org/cgi-bin/mature_mir.cgi?name=hsa-miR-155-5p) | submission |
| 6 | 87 | [hsa-miR-1199-5p](https://mirdb.org/cgi-bin/mature_mir.cgi?name=hsa-miR-1199-5p) | submission |
| 7 | 86 | [hsa-miR-200b-5p](https://mirdb.org/cgi-bin/mature_mir.cgi?name=hsa-miR-200b-5p) | submission |
| 8 | 86 | [hsa-miR-200a-5p](https://mirdb.org/cgi-bin/mature_mir.cgi?name=hsa-miR-200a-5p) | submission |
| 9 | 84 | [hsa-miR-216a-5p](https://mirdb.org/cgi-bin/mature_mir.cgi?name=hsa-miR-216a-5p) | submission |
| 10 | 83 | [hsa-miR-4310](https://mirdb.org/cgi-bin/mature_mir.cgi?name=hsa-miR-4310) | submission |
| 11 | 80 | [hsa-miR-548c-3p](https://mirdb.org/cgi-bin/mature_mir.cgi?name=hsa-miR-548c-3p) | submission |
| 12 | 80 | [hsa-miR-7157-5p](https://mirdb.org/cgi-bin/mature_mir.cgi?name=hsa-miR-7157-5p) | submission |
| 13 | 79 | [hsa-miR-449b-3p](https://mirdb.org/cgi-bin/mature_mir.cgi?name=hsa-miR-449b-3p) | submission |
| 14 | 79 | [hsa-miR-4303](https://mirdb.org/cgi-bin/mature_mir.cgi?name=hsa-miR-4303) | submission |
| 15 | 77 | [hsa-miR-3658](https://mirdb.org/cgi-bin/mature_mir.cgi?name=hsa-miR-3658) | submission |
| 16 | 77 | [hsa-miR-580-5p](https://mirdb.org/cgi-bin/mature_mir.cgi?name=hsa-miR-580-5p) | submission |
| 17 | 76 | [hsa-miR-7850-5p](https://mirdb.org/cgi-bin/mature_mir.cgi?name=hsa-miR-7850-5p) | submission |
| 18 | 75 | [hsa-miR-449c-5p](https://mirdb.org/cgi-bin/mature_mir.cgi?name=hsa-miR-449c-5p) | submission |
| **19** | **75** | [**hsa-miR-34b-5p**](https://mirdb.org/cgi-bin/mature_mir.cgi?name=hsa-miR-34b-5p) | **submission** |
| 20 | 74 | [hsa-let-7c-3p](https://mirdb.org/cgi-bin/mature_mir.cgi?name=hsa-let-7c-3p) | submission |
| 21 | 73 | [hsa-miR-590-3p](https://mirdb.org/cgi-bin/mature_mir.cgi?name=hsa-miR-590-3p) | submission |
| 22 | 72 | [hsa-miR-4328](https://mirdb.org/cgi-bin/mature_mir.cgi?name=hsa-miR-4328) | submission |
| 23 | 72 | [hsa-miR-1277-5p](https://mirdb.org/cgi-bin/mature_mir.cgi?name=hsa-miR-1277-5p) | submission |
| 24 | 72 | [hsa-miR-1250-3p](https://mirdb.org/cgi-bin/mature_mir.cgi?name=hsa-miR-1250-3p) | submission |
| 25 | 72 | [hsa-miR-202-3p](https://mirdb.org/cgi-bin/mature_mir.cgi?name=hsa-miR-202-3p) | submission |
| 26 | 72 | [hsa-miR-4503](https://mirdb.org/cgi-bin/mature_mir.cgi?name=hsa-miR-4503) | submission |
| 27 | 71 | [hsa-miR-30e-5p](https://mirdb.org/cgi-bin/mature_mir.cgi?name=hsa-miR-30e-5p) | submission |
| 28 | 71 | [hsa-miR-30d-5p](https://mirdb.org/cgi-bin/mature_mir.cgi?name=hsa-miR-30d-5p) | submission |
| 29 | 71 | [hsa-miR-30c-5p](https://mirdb.org/cgi-bin/mature_mir.cgi?name=hsa-miR-30c-5p) | submission |
| 30 | 71 | [hsa-miR-30b-5p](https://mirdb.org/cgi-bin/mature_mir.cgi?name=hsa-miR-30b-5p) | submission |
| 31 | 71 | [hsa-miR-30a-5p](https://mirdb.org/cgi-bin/mature_mir.cgi?name=hsa-miR-30a-5p) | submission |
| 32 | 70 | [hsa-miR-3671](https://mirdb.org/cgi-bin/mature_mir.cgi?name=hsa-miR-3671) | submission |
| 33 | 69 | [hsa-miR-2682-5p](https://mirdb.org/cgi-bin/mature_mir.cgi?name=hsa-miR-2682-5p) | submission |
| 34 | 69 | [hsa-miR-335-3p](https://mirdb.org/cgi-bin/mature_mir.cgi?name=hsa-miR-335-3p) | submission |
| 35 | 69 | [hsa-miR-3972](https://mirdb.org/cgi-bin/mature_mir.cgi?name=hsa-miR-3972) | submission |
| 36 | 69 | [hsa-miR-1202](https://mirdb.org/cgi-bin/mature_mir.cgi?name=hsa-miR-1202) | submission |
| 37 | 68 | [hsa-miR-4775](https://mirdb.org/cgi-bin/mature_mir.cgi?name=hsa-miR-4775) | submission |
| 38 | 68 | [hsa-miR-1324](https://mirdb.org/cgi-bin/mature_mir.cgi?name=hsa-miR-1324) | submission |
| 39 | 67 | [hsa-miR-7-2-3p](https://mirdb.org/cgi-bin/mature_mir.cgi?name=hsa-miR-7-2-3p) | submission |
| 40 | 67 | [hsa-miR-7-1-3p](https://mirdb.org/cgi-bin/mature_mir.cgi?name=hsa-miR-7-1-3p) | submission |
| 41 | 67 | [hsa-miR-23c](https://mirdb.org/cgi-bin/mature_mir.cgi?name=hsa-miR-23c) | submission |
| 42 | 67 | [hsa-miR-23b-3p](https://mirdb.org/cgi-bin/mature_mir.cgi?name=hsa-miR-23b-3p) | submission |
| 43 | 67 | [hsa-miR-23a-3p](https://mirdb.org/cgi-bin/mature_mir.cgi?name=hsa-miR-23a-3p) | submission |
| 44 | 65 | [hsa-miR-4795-3p](https://mirdb.org/cgi-bin/mature_mir.cgi?name=hsa-miR-4795-3p) | submission |
| 45 | 65 | [hsa-miR-3190-3p](https://mirdb.org/cgi-bin/mature_mir.cgi?name=hsa-miR-3190-3p) | submission |
| 46 | 65 | [hsa-miR-5087](https://mirdb.org/cgi-bin/mature_mir.cgi?name=hsa-miR-5087) | submission |
| 47 | 64 | [hsa-miR-567](https://mirdb.org/cgi-bin/mature_mir.cgi?name=hsa-miR-567) | submission |
| 48 | 63 | [hsa-miR-4999-5p](https://mirdb.org/cgi-bin/mature_mir.cgi?name=hsa-miR-4999-5p) | submission |
| 49 | 62 | [hsa-miR-8055](https://mirdb.org/cgi-bin/mature_mir.cgi?name=hsa-miR-8055) | submission |
| 50 | 61 | [hsa-miR-491-3p](https://mirdb.org/cgi-bin/mature_mir.cgi?name=hsa-miR-491-3p) | submission |
| 51 | 61 | [hsa-miR-192-3p](https://mirdb.org/cgi-bin/mature_mir.cgi?name=hsa-miR-192-3p) | submission |
| 52 | 60 | [hsa-miR-12122](https://mirdb.org/cgi-bin/mature_mir.cgi?name=hsa-miR-12122) | submission |
| 53 | 59 | [hsa-miR-5692a](https://mirdb.org/cgi-bin/mature_mir.cgi?name=hsa-miR-5692a) | submission |
| 54 | 58 | [hsa-miR-2053](https://mirdb.org/cgi-bin/mature_mir.cgi?name=hsa-miR-2053) | submission |
| 55 | 55 | [hsa-miR-7159-3p](https://mirdb.org/cgi-bin/mature_mir.cgi?name=hsa-miR-7159-3p) | submission |
| 56 | 54 | [hsa-miR-4530](https://mirdb.org/cgi-bin/mature_mir.cgi?name=hsa-miR-4530) | submission |
| 57 | 54 | [hsa-miR-501-5p](https://mirdb.org/cgi-bin/mature_mir.cgi?name=hsa-miR-501-5p) | submission |
| 58 | 54 | [hsa-miR-130a-5p](https://mirdb.org/cgi-bin/mature_mir.cgi?name=hsa-miR-130a-5p) | submission |
| 59 | 52 | [hsa-miR-7856-5p](https://mirdb.org/cgi-bin/mature_mir.cgi?name=hsa-miR-7856-5p) | submission |
| 60 | 52 | [hsa-miR-944](https://mirdb.org/cgi-bin/mature_mir.cgi?name=hsa-miR-944) | submission |
| 61 | 52 | [hsa-miR-4517](https://mirdb.org/cgi-bin/mature_mir.cgi?name=hsa-miR-4517) | submission |
| 62 | 51 | [hsa-miR-4520-2-3p](https://mirdb.org/cgi-bin/mature_mir.cgi?name=hsa-miR-4520-2-3p) | submission |
| 63 | 51 | [hsa-miR-4735-5p](https://mirdb.org/cgi-bin/mature_mir.cgi?name=hsa-miR-4735-5p) | submission |
| 64 | 51 | [hsa-miR-5692c](https://mirdb.org/cgi-bin/mature_mir.cgi?name=hsa-miR-5692c) | submission |
| 65 | 51 | [hsa-miR-5692b](https://mirdb.org/cgi-bin/mature_mir.cgi?name=hsa-miR-5692b) | submission |
| 66 | 51 | [hsa-miR-569](https://mirdb.org/cgi-bin/mature_mir.cgi?name=hsa-miR-569) | submission |
| 67 | 50 | [hsa-miR-4319](https://mirdb.org/cgi-bin/mature_mir.cgi?name=hsa-miR-4319) | submission |
