# Supplementary material for: lncRNA HIF1A-AS2 acts as an oncogene to regulate malignant phenotypes in cervical cancer
Source: Front Oncol. 2025 Feb 27;15:1530677. doi: 10.3389/fonc.2025.1530677 (PMC11912943; doi:10.3389/fonc.2025.1530677)
Supplement: Supplementary file 9 [file Table4.docx]

Table IV. The 50 predicted targets for miR-34b-5p.

| Target Rank | Target Score | miRNA Name | Gene Symbol | Gene Description |
| --- | --- | --- | --- | --- |
| 1 | 100 | hsa-miR-34b-5p | [TENM1](http://www.ncbi.nlm.nih.gov/entrez/query.fcgi?db=gene&cmd=Retrieve&dopt=full_report&list_uids=10178) | teneurin transmembrane protein 1 |
| 2 | 98 | hsa-miR-34b-5p | [ELMOD1](http://www.ncbi.nlm.nih.gov/entrez/query.fcgi?db=gene&cmd=Retrieve&dopt=full_report&list_uids=55531) | ELMO domain containing 1 |
| 3 | 98 | hsa-miR-34b-5p | [RFX3](http://www.ncbi.nlm.nih.gov/entrez/query.fcgi?db=gene&cmd=Retrieve&dopt=full_report&list_uids=5991) | regulatory factor X3 |
| 4 | 97 | hsa-miR-34b-5p | [ZC4H2](http://www.ncbi.nlm.nih.gov/entrez/query.fcgi?db=gene&cmd=Retrieve&dopt=full_report&list_uids=55906) | zinc finger C4H2-type containing |
| 5 | 97 | hsa-miR-34b-5p | [RAB3C](http://www.ncbi.nlm.nih.gov/entrez/query.fcgi?db=gene&cmd=Retrieve&dopt=full_report&list_uids=115827) | RAB3C, member RAS oncogene family |
| 6 | 97 | hsa-miR-34b-5p | [DLL1](http://www.ncbi.nlm.nih.gov/entrez/query.fcgi?db=gene&cmd=Retrieve&dopt=full_report&list_uids=28514) | delta like canonical Notch ligand 1 |
| 7 | 96 | hsa-miR-34b-5p | [PLEKHA1](http://www.ncbi.nlm.nih.gov/entrez/query.fcgi?db=gene&cmd=Retrieve&dopt=full_report&list_uids=59338) | pleckstrin homology domain containing A1 |
| 8 | 96 | hsa-miR-34b-5p | [THRB](http://www.ncbi.nlm.nih.gov/entrez/query.fcgi?db=gene&cmd=Retrieve&dopt=full_report&list_uids=7068) | thyroid hormone receptor beta |
| 9 | 96 | hsa-miR-34b-5p | [CAMSAP2](http://www.ncbi.nlm.nih.gov/entrez/query.fcgi?db=gene&cmd=Retrieve&dopt=full_report&list_uids=23271) | calmodulin regulated spectrin associated protein family member 2 |
| 10 | 96 | hsa-miR-34b-5p | [STK38L](http://www.ncbi.nlm.nih.gov/entrez/query.fcgi?db=gene&cmd=Retrieve&dopt=full_report&list_uids=23012) | serine/threonine kinase 38 like |
| 11 | 95 | hsa-miR-34b-5p | [PIK3C2A](http://www.ncbi.nlm.nih.gov/entrez/query.fcgi?db=gene&cmd=Retrieve&dopt=full_report&list_uids=5286) | phosphatidylinositol-4-phosphate 3-kinase catalytic subunit type 2 alpha |
| 12 | 95 | hsa-miR-34b-5p | [NEUROD1](http://www.ncbi.nlm.nih.gov/entrez/query.fcgi?db=gene&cmd=Retrieve&dopt=full_report&list_uids=4760) | neuronal differentiation 1 |
| 13 | 95 | hsa-miR-34b-5p | [ANKS1B](http://www.ncbi.nlm.nih.gov/entrez/query.fcgi?db=gene&cmd=Retrieve&dopt=full_report&list_uids=56899) | ankyrin repeat and sterile alpha motif domain containing 1B |
| 14 | 95 | hsa-miR-34b-5p | [SOX6](http://www.ncbi.nlm.nih.gov/entrez/query.fcgi?db=gene&cmd=Retrieve&dopt=full_report&list_uids=55553) | SRY-box 6 |
| 15 | 94 | hsa-miR-34b-5p | [ASCL1](http://www.ncbi.nlm.nih.gov/entrez/query.fcgi?db=gene&cmd=Retrieve&dopt=full_report&list_uids=429) | achaete-scute family bHLH transcription factor 1 |
| 16 | 93 | hsa-miR-34b-5p | [ELMSAN1](http://www.ncbi.nlm.nih.gov/entrez/query.fcgi?db=gene&cmd=Retrieve&dopt=full_report&list_uids=91748) | ELM2 and Myb/SANT domain containing 1 |
| 17 | 93 | hsa-miR-34b-5p | [APH1A](http://www.ncbi.nlm.nih.gov/entrez/query.fcgi?db=gene&cmd=Retrieve&dopt=full_report&list_uids=51107) | aph-1 homolog A, gamma-secretase subunit |
| 18 | 93 | hsa-miR-34b-5p | [THAP12](http://www.ncbi.nlm.nih.gov/entrez/query.fcgi?db=gene&cmd=Retrieve&dopt=full_report&list_uids=5612) | THAP domain containing 12 |
| Continue |  |  |  |  |
| 19 | 93 | hsa-miR-34b-5p | [ATP11C](http://www.ncbi.nlm.nih.gov/entrez/query.fcgi?db=gene&cmd=Retrieve&dopt=full_report&list_uids=286410) | ATPase phospholipid transporting 11C |
| **20** | **93** | **hsa-miR-34b-5p** | [**RDX**](http://www.ncbi.nlm.nih.gov/entrez/query.fcgi?db=gene&cmd=Retrieve&dopt=full_report&list_uids=5962) | **radixin** |
| 21 | 93 | hsa-miR-34b-5p | [MYF5](http://www.ncbi.nlm.nih.gov/entrez/query.fcgi?db=gene&cmd=Retrieve&dopt=full_report&list_uids=4617) | myogenic factor 5 |
| 22 | 93 | hsa-miR-34b-5p | [ARID1B](http://www.ncbi.nlm.nih.gov/entrez/query.fcgi?db=gene&cmd=Retrieve&dopt=full_report&list_uids=57492) | AT-rich interaction domain 1B |
| 23 | 93 | hsa-miR-34b-5p | [GAS1](http://www.ncbi.nlm.nih.gov/entrez/query.fcgi?db=gene&cmd=Retrieve&dopt=full_report&list_uids=2619) | growth arrest specific 1 |
| 24 | 93 | hsa-miR-34b-5p | [CBLB](http://www.ncbi.nlm.nih.gov/entrez/query.fcgi?db=gene&cmd=Retrieve&dopt=full_report&list_uids=868) | Cbl proto-oncogene B |
| 25 | 92 | hsa-miR-34b-5p | [HOXB8](http://www.ncbi.nlm.nih.gov/entrez/query.fcgi?db=gene&cmd=Retrieve&dopt=full_report&list_uids=3218) | homeobox B8 |
| 26 | 92 | hsa-miR-34b-5p | [MARVELD2](http://www.ncbi.nlm.nih.gov/entrez/query.fcgi?db=gene&cmd=Retrieve&dopt=full_report&list_uids=153562) | MARVEL domain containing 2 |
| 27 | 92 | hsa-miR-34b-5p | [KCNA1](http://www.ncbi.nlm.nih.gov/entrez/query.fcgi?db=gene&cmd=Retrieve&dopt=full_report&list_uids=3736) | potassium voltage-gated channel subfamily A member 1 |
| 28 | 92 | hsa-miR-34b-5p | [MYCBP2](http://www.ncbi.nlm.nih.gov/entrez/query.fcgi?db=gene&cmd=Retrieve&dopt=full_report&list_uids=23077) | MYC binding protein 2, E3 ubiquitin protein ligase |
| 29 | 92 | hsa-miR-34b-5p | [ATP6V0A2](http://www.ncbi.nlm.nih.gov/entrez/query.fcgi?db=gene&cmd=Retrieve&dopt=full_report&list_uids=23545) | ATPase H+ transporting V0 subunit a2 |
| 30 | 92 | hsa-miR-34b-5p | [STK39](http://www.ncbi.nlm.nih.gov/entrez/query.fcgi?db=gene&cmd=Retrieve&dopt=full_report&list_uids=27347) | serine/threonine kinase 39 |
| 31 | 92 | hsa-miR-34b-5p | [QDPR](http://www.ncbi.nlm.nih.gov/entrez/query.fcgi?db=gene&cmd=Retrieve&dopt=full_report&list_uids=5860) | quinoid dihydropteridine reductase |
| 32 | 92 | hsa-miR-34b-5p | [CDK19](http://www.ncbi.nlm.nih.gov/entrez/query.fcgi?db=gene&cmd=Retrieve&dopt=full_report&list_uids=23097) | cyclin dependent kinase 19 |
| 33 | 92 | hsa-miR-34b-5p | [CNTNAP1](http://www.ncbi.nlm.nih.gov/entrez/query.fcgi?db=gene&cmd=Retrieve&dopt=full_report&list_uids=8506) | contactin associated protein 1 |
| 34 | 92 | hsa-miR-34b-5p | [HOXC8](http://www.ncbi.nlm.nih.gov/entrez/query.fcgi?db=gene&cmd=Retrieve&dopt=full_report&list_uids=3224) | homeobox C8 |
| 35 | 91 | hsa-miR-34b-5p | [PHACTR1](http://www.ncbi.nlm.nih.gov/entrez/query.fcgi?db=gene&cmd=Retrieve&dopt=full_report&list_uids=221692) | phosphatase and actin regulator 1 |
| 36 | 91 | hsa-miR-34b-5p | [MYC](http://www.ncbi.nlm.nih.gov/entrez/query.fcgi?db=gene&cmd=Retrieve&dopt=full_report&list_uids=4609) | MYC proto-oncogene, bHLH transcription factor |
| 37 | 91 | hsa-miR-34b-5p | [CTNND2](http://www.ncbi.nlm.nih.gov/entrez/query.fcgi?db=gene&cmd=Retrieve&dopt=full_report&list_uids=1501) | catenin delta 2 |
| 38 | 91 | hsa-miR-34b-5p | [APOB](http://www.ncbi.nlm.nih.gov/entrez/query.fcgi?db=gene&cmd=Retrieve&dopt=full_report&list_uids=338) | apolipoprotein B |
| 39 | 91 | hsa-miR-34b-5p | [STMN2](http://www.ncbi.nlm.nih.gov/entrez/query.fcgi?db=gene&cmd=Retrieve&dopt=full_report&list_uids=11075) | stathmin 2 |
| 40 | 91 | hsa-miR-34b-5p | [MTCL1](http://www.ncbi.nlm.nih.gov/entrez/query.fcgi?db=gene&cmd=Retrieve&dopt=full_report&list_uids=23255) | microtubule crosslinking factor 1 |
| 41 | 91 | hsa-miR-34b-5p | [NHSL1](http://www.ncbi.nlm.nih.gov/entrez/query.fcgi?db=gene&cmd=Retrieve&dopt=full_report&list_uids=57224) | NHS like 1 |
| 42 | 90 | hsa-miR-34b-5p | [CELF2](http://www.ncbi.nlm.nih.gov/entrez/query.fcgi?db=gene&cmd=Retrieve&dopt=full_report&list_uids=10659) | CUGBP Elav-like family member 2 |
| 43 | 90 | hsa-miR-34b-5p | [FKBP1B](http://www.ncbi.nlm.nih.gov/entrez/query.fcgi?db=gene&cmd=Retrieve&dopt=full_report&list_uids=2281) | FKBP prolyl isomerase 1B |
| 44 | 90 | hsa-miR-34b-5p | [PIEZO2](http://www.ncbi.nlm.nih.gov/entrez/query.fcgi?db=gene&cmd=Retrieve&dopt=full_report&list_uids=63895) | piezo type mechanosensitive ion channel component 2 |
| 45 | 90 | hsa-miR-34b-5p | [CADM2](http://www.ncbi.nlm.nih.gov/entrez/query.fcgi?db=gene&cmd=Retrieve&dopt=full_report&list_uids=253559) | cell adhesion molecule 2 |
| 46 | 90 | hsa-miR-34b-5p | [XKR6](http://www.ncbi.nlm.nih.gov/entrez/query.fcgi?db=gene&cmd=Retrieve&dopt=full_report&list_uids=286046) | XK related 6 |
| 47 | 90 | hsa-miR-34b-5p | [WIPF3](http://www.ncbi.nlm.nih.gov/entrez/query.fcgi?db=gene&cmd=Retrieve&dopt=full_report&list_uids=644150) | WAS/WASL interacting protein family member 3 |
| 48 | 90 | hsa-miR-34b-5p | [MAPK4](http://www.ncbi.nlm.nih.gov/entrez/query.fcgi?db=gene&cmd=Retrieve&dopt=full_report&list_uids=5596) | mitogen-activated protein kinase 4 |
| 49 | 90 | hsa-miR-34b-5p | [RALA](http://www.ncbi.nlm.nih.gov/entrez/query.fcgi?db=gene&cmd=Retrieve&dopt=full_report&list_uids=5898) | RAS like proto-oncogene A |
| 50 | 90 | hsa-miR-34b-5p | [MTDH](http://www.ncbi.nlm.nih.gov/entrez/query.fcgi?db=gene&cmd=Retrieve&dopt=full_report&list_uids=92140) | metadherin |

There are 570 predicted targets for hsa-miR-34b-5p in miRDB. Due to the large amount data, the table shows only 50 data.
