# Supplementary material for: lncRNA HIF1A-AS2 acts as an oncogene to regulate malignant phenotypes in cervical cancer
Source: Front Oncol. 2025 Feb 27;15:1530677. doi: 10.3389/fonc.2025.1530677 (PMC11912943; doi:10.3389/fonc.2025.1530677)
Supplement: Supplementary file 10 [file Table5.docx]

Table SV. The clinical characteristic of cervical cancer patients.

| Number | Age | Type | Clinical phase |
| --- | --- | --- | --- |
| 1 | 58 | squamous cell carcinoma | ⅠA |
| 2 | 49 | squamous cell carcinoma | ⅠB2 |
| 3 | 46 | squamous cell carcinoma | ⅠB2 |
| 4 | 66 | squamous cell carcinoma | ⅡA2 |
| 5 | 48 | squamous cell carcinoma | ⅠB2 |
| 6 | 59 | squamous cell carcinoma | ⅠA |
| 7 | 57 | adenocarcinomas | ⅡA1 |
| 8 | 63 | adenocarcinomas | ⅠA |
| 9 | 48 | squamous cell carcinoma | ⅠB2 |
| 10 | 50 | squamous cell carcinoma | ⅠB |
| 11 | 42 | squamous cell carcinoma | ⅡB |
| 12 | 41 | squamous cell carcinoma | ⅠB |
| 13 | 38 | squamous cell carcinoma | ⅠB2 |
| 14 | 52 | squamous cell carcinoma | ⅠB1 |
| 15 | 45 | squamous cell carcinoma | ⅡB |
| 16 | 36 | squamous cell carcinoma | ⅠB1 |
| 17 | 56 | squamous cell carcinoma | ⅠB2 |
| 18 | 50 | squamous cell carcinoma | ⅠB2 |
| 19 | 47 | squamous cell carcinoma | ⅡB |
| 20 | 56 | squamous cell carcinoma | ⅡA2 |
