# Supplementary material for: lncRNA HIF1A-AS2 acts as an oncogene to regulate malignant phenotypes in cervical cancer
Source: Front Oncol. 2025 Feb 27;15:1530677. doi: 10.3389/fonc.2025.1530677 (PMC11912943; doi:10.3389/fonc.2025.1530677)
Supplement: Supplementary file 11 [file Table6.docx]

Table SVI. The primer sequences used in this study.

| Primers | Primer sequences (5’-3’) |
| --- | --- |
| **RT-qPCR** |  |
| β-actin: forward | GGCGGCACCACCATGTACCCT |
| β-actin: reverse | AGGGGCCGGACTCGTCATACT |
| HIF1A-AS2: forward | ATGTAGGAAGTGCCAGAGCC |
| HIF1A-AS2: reverse | TCTGTGGCTCAGTTCCTTTTGT |
| radixin: forward | AATGCCGAAACCAATCAACG |
| radixin: reverse | ACCTCACGCAAACCAACTGT |
| **ChIp Assay** |  |
| P2-HIF1A-AS2-1: forward | GAGCTTGTTGTCTTAGTGACTG |
| P2-HIF1A-AS2-1: reverse | TATGTAATGACCAGGAGTTCAGG |
| P2-HIF1A-AS2-2: forward | AGGATTATAGTTGCCAGGGTTG |
| P2-HIF1A-AS2-2: reverse | ACCCACTGCTCAGTCAC |
| P2-HIF1A-AS2-3: forward | GGAAGGGCCAGTTCTGC |
| P2-HIF1A-AS2-3: reverse | TTGAGAAGGGAAAGCCACG |
| P2-HIF1A-AS2-4: forward | GCTTTCCCTTCTCAATGCTTTC |
| P2-HIF1A-AS2-4: reverse | CTGTGACCCAAAGTGCTTC |
| P2-HIF1A-AS2-5: forward | CTTTAACGTGGCTTTCCCTTC |
| P2-HIF1A-AS2-5: reverse | CACAAACACTGTGACCCAAAG |
| P2-HIF1A-AS2-6: forward | GCTGTACTGTCCTTTAACGTG |
| P2-HIF1A-AS2-6: reverse | TTAAGCACAAACACTGTGACC |
| **RIP Assay** |  |
| HIF1A-AS2: forward | GCTACTGCAATGCAATGGTTT |
| HIF1A-AS2: reverse | TGGACACTGGTGGCTCATTA |
| miR-34b-5p-RT | CTCAACTGGTGTCGTGGAGTCGGCAATTCAGTTGAGCAATCAGC |
| miR-34b-5p: forward | ACACTCCAGCTGGGTAGGCAGTGTCATTAGC |
| miR-34b-5p: reverse | TGGTGTCGTGGAGTCG |
